# Antiaging Metabolite‐Based Polymeric Microparticles for Intracellular Drug Delivery and Bone Regeneration

**DOI:** 10.1002/smsc.202400201

**Published:** 2024-09-03

**Authors:** Zhuozhi Wang, Jue Hu, Jeffrey S. Marschall, Ling Yang, Erliang Zeng, Shaoping Zhang, Hongli Sun

**Affiliations:** ^1^ Iowa Institute for Oral Health Research University of Iowa College of Dentistry Iowa City IA 52242 USA; ^2^ Department of Oral and Maxillofacial Surgery University of Iowa College of Dentistry Iowa City IA 52242 USA; ^3^ Department of Anatomy and Cell Biology, Fraternal Order of Eagles Diabetes Research Center, Pappajohn Biomedical Institute University of Iowa Carver College of Medicine Iowa City IA 52242 USA; ^4^ Division of Biostatistics and Computational Biology University of Iowa College of Dentistry Iowa City IA 52242 USA; ^5^ Department of Periodontics University of Iowa College of Dentistry Iowa City IA 52242 USA; ^6^ Roy J. Carver Department of Biomedical Engineering University of Iowa College of Engineering Iowa City IA 52242 USA

**Keywords:** α‐ketoglutarate, bone regeneration, intracellular drug delivery, microparticle, osteogenic differentiation

## Abstract

α‐ketoglutarate (AKG), a key component of the tricarboxylic acid cycle, has attracted attention for its antiaging properties. In the recent study, it is indicated that locally delivered cell‐permeable AKG significantly promotes osteogenic differentiation and mouse bone regeneration. However, the cytotoxicity and rapid hydrolysis of the metabolite limit its application. In this study, novel AKG‐based polymeric microparticles (PAKG MPs) are synthesized for sustained release. In vitro data suggest that the chemical components, hydrophilicity, and size of the MPs can significantly affect their cytotoxicity and pro‐osteogenic activity. Excitingly, these biodegradable PAKG MPs are highly phagocytosable for nonphagocytic pre‐osteoblasts MC3T3‐E1 and primary bone marrow mesenchymal stem cells, significantly promoting their osteoblastic differentiation. RNA‐Sequencing (RNA‐Seq) data suggest that PAKG MPs strongly activate Wnt/β‐catenin and PI3K–Akt pathways for osteogenic differentiation. Moreover, PAKG enables poly(L‐lactic acid) and poly(lactic*‐co*‐glycolic acid) MPs (PLGA MPs) for efficient phagocytosis. In this data, it is indicated that PLGA–PAKG‐MPs‐mediated intracellular drug delivery can significantly promote stronger osteoblastic differentiation compared to PLGA‐MPs‐delivered phenamil. Notably, PAKG MPs significantly improve large bone regeneration in a mouse cranial bone defect model. Thus, the novel PAKG‐based MPs show great promise to improve osteogenic differentiation and bone regeneration and enable efficient intracellular drug delivery for broad regenerative medicine.

## Graphical abstract

Novel metabolite α‐ketoglutarate‐based polymeric microparticles (PAKG MPs) are synthesized for bone tissue regeneration. Unlike conventional biodegradable MPs, PAKG MPs are effectively taken up by osteogenic cells, promoting their differentiation and enhancing cranial bone regeneration in a mouse model.

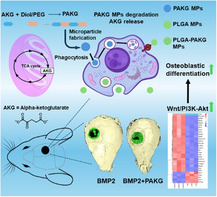

## 1 Introduction

Reconstruction of large bone defects caused by trauma, congenital anomalies, or pathology remains a clinical challenge. Although autologous tissue remains the gold standard, its application is impeded by limited availability and donor site morbidity.^[^ ^1^, ^2^ ^]^ Biomaterial‐based tissue engineering presents an alternative strategy to address the challenges posed by autologous reconstruction. Biomaterial‐mediated delivery of growth factors, such as bone morphogenetic protein 2 (BMP2), and transplantation of stem cells are an intensively studied approach for promoting bone regeneration.^[^ ^3^, ^4^, ^5^, ^6^ ^]^ However, the low efficacy of BMP2 necessitates high doses, which can lead to serious side effects. Thus, innovative biomaterials with inherent pro‐osteogenic properties are particularly promising for bone tissue engineering. These materials can potentially reduce the dose of BMP2, thereby minimizing potential side effects. Similarly, stem cells transplantation is fraught with challenges, including potential immunological rejection^[^ ^7^, ^8^ ^]^ and the elevated risk of tumorigenesis.^[^ ^9^, ^10^, ^11^ ^]^ Therefore, a need exists to develop innovative biomaterials with robust pro‐osteogenic properties, thereby reducing reliance on these intricate biological mediators.

α‐ketoglutarate (αKG or AKG) is emerging as an intriguing endogenous antiaging molecule across various organisms. As an essential tricarboxylic acid (TCA) cycle intermediate and an energy donor, AKG was first reported to extend the lifespan of adult *Caenorhabditis elegans* by ≈50% in 2014.^[^ ^12^ ^]^ Furthermore, another study revealed that dietary AKG could extend Drosophila lifespan by modulating mammalian target of rapamycin (mTOR)/AMP‐activated protein kinase pathways.^[^ ^13^ ^]^ A recent study indicated that dietary AKG can significantly expand the lifespan and healthspan of aged mice in 2020.^[^ ^14^ ^]^ While AKG's specific role in bone formation remains unclear, it is recognized as an essential metabolite for bone anabolism.^[^ ^15^, ^16^, ^17^, ^18^ ^]^ For example, dietary AKG is known for supporting amino acid and collagen matrix synthesis.^[^ ^19^, ^20^ ^]^ The enzyme glutaminase and AKG production via glutamine metabolism were found essential for skeletal stem cells.^[^ ^21^ ^]^ Additionally, AKG production plays a crucial role in parathyroid hormone^[^ ^22^ ^]^ and wingless‐related integration site (Wnt)^[^ ^23^ ^]^‐induced bone anabolism. While circulatory AKG levels significantly decrease in older humans and mice,^[^ ^24^, ^25^ ^]^ dietary supplementation with AKG increases circulating AKG levels in aged mice, mitigates age‐related osteoporosis, and promotes new bone formation.^[^ ^26^ ^]^ Taken together, AKG has shown great promise in promoting bone regeneration.

The therapeutic applicability of locally applied AKG for bone regeneration is currently hindered due to poor cell permeability and lack of biomaterial‐based AKG delivery. Our recent investigation indicates that a cell‐permeable AKG derivative (dimethyl‐AKG, DMAKG) can promote osteoblastic differentiation of mouse cranial pre‐osteoblastic cells and primary bone marrow mesenchymal stem cells (BMSCs). More importantly, for the first time, our in vivo study indicates that local scaffold (gelfoam)‐released DMAKG significantly improves BMP2‐induced ectopic bone regeneration in both young and aged mice.^[^ ^27^ ^]^ Despite this, it is still challenging to directly apply the soluble AKG or DMAKG for tissue regeneration due to low stability in an aqueous environment and poor bioavailability. Remarkably, akin to other metabolites like lactic acid and citric acid, AKG can undergo polymerization, resulting in the formation of biodegradable polyesters. When these polyesters are shaped into macroparticles (MPs), they exhibit the capacity to modulate not only the immune response of dendritic cells (DCs) but also the metabolic processes and innate immune cell phenotypes (specifically macrophages and neutrophils) through phagocytosis.^[^ ^28^, ^29^, ^30^ ^]^ However, it's important to note that this phagocytosis‐mediated intracellular delivery strategy may not be applicable to nonphagocytic cells (such as osteoblasts and MSCs), which have limited MPs uptake capability. Nevertheless, this implies that the AKG‐based biopolymer holds promise as an innovative approach for creating biodegradable biomaterials specifically tailored for osteogenesis and bone regeneration, an area that remains largely unexplored.

Inspired by its promising reparative abilities, we designed and synthesized innovative polyesters containing AKG monomer and used them to fabricate biodegradable MPs (PAKG MPs) for bone tissue engineering applications in this study. Through tailoring the chemical composition of the copolymers and the particle size of PAKG MPs, we can significantly reduce the cytotoxicity to pre‐osteoblasts and promote their osteoblastic differentiation and matrix mineralization. While MPs made by common polyesters, e.g., poly(L‐lactic acid) and poly(lactic*‐co*‐glycolic acid) (PLLA and PLGA MPs) have low uptake by osteoblastic cells, our innovative PAKG MPs are highly phagocytosable for both pre‐osteoblastic cells and BMSCs. Furthermore, blending with 1% of PAKG enables these PLLA‐ or PLGA MPs‐efficient phagocytosis for intracellular drug delivery. Using the BMP agonist, phenamil^[^ ^31^, ^32^, ^33^ ^]^ as a model small‐molecule drug, our data indicate that PLGA–PAKG‐MP‐mediated intracellular drug delivery can promote significantly faster and stronger osteoblastic differentiation and matrix mineralization compared to free or conventional PLGA‐MPs‐delivered phenamil groups. Our in vivo data confirm that PAKG MPs significantly improve bone regeneration in a mouse cranial defect model.

## 2 Results

### 2.1 Synthesis and Characterization of PAKG Polymers and PAKG MPs

**Figure 1:**
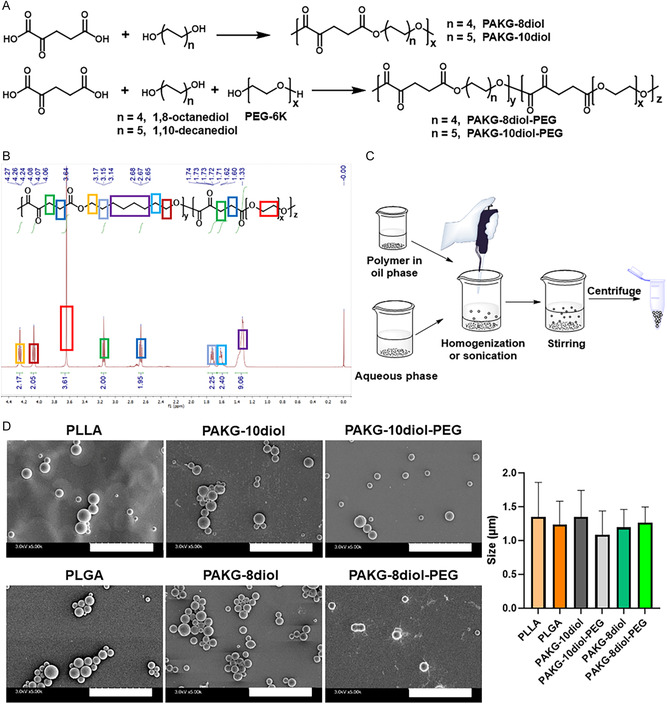
AKG polymer (PAKG) synthesis, characterization, and microparticles (MPs) fabrication, characterization. A) Scheme of PAKG‐10diol, PAKG–10diol–PEG, PAKG–8diol, and PAKG–8diol–PEG synthesis. B) ^1^H NMR of PAKG–8diol–PEG. C) Schematic illustration of the fabrication of PAKG MPs. D) Scanning electron microscope (SEM) images of PLLA, PLGA, PAKG‐10diol, PAKG‐10diol‐PEG, PAKG‐8diol, and PAKG‐8diol‐PEG MPs (fabricated by homogenization) and their size distribution (scale bars = 10 μm).

The polyesters composed of AKG and 1,8‐octanediol/1,10‐decanediol with or without polyethylene glycol (PEG) were synthesized through polycondensation without solvent by melting all reactants at 120 °C (**Figure** **1**A). The side product, water, was removed from the reaction system simultaneously by vacuum to drive the reaction toward polymer formation. The molecular weight (Mw) of the four polymers were evaluated with gel permeation chromatography and they are 12 431, 11 365, 24 919, and 12 351 g mol^−1^ with polydispersity index 1.79, 1.12, 2.17, and 2.36 for PAKG–10diol, PAKG–8diol, PAKG–10diol–PEG, and PAKG‐8diol‐PEG, respectively. The four polymers were then characterized by proton nuclear magnetic resonance (1H NMR) to confirm the structure and determine the ratio of PEG in polymers (Figure 1B and Figure S1–S3, Supporting Information). The two triplet peaks near 2.7 and 3.2 ppm corresponded to two —CH_2_— groups in AKG while the two triplet peaks near 4.1 and 4.3 ppm corresponded to two end —CH_2_— groups adjacent to O in diol for all four polymers. The three peaks between 1.2 and 1.8 ppm corresponded to the —CH_2_— groups between two end —CH_2_— groups in diol. For the two polymers containing PEG, the peak near 3.65 ppm corresponded to —CH_2_— groups in PEG. Therefore, according to the integration of this PEG peak, the weight ratio of PEG could be calculated to be 22% for PAKG–10diol–PEG and 13.4% for PAKG–8diol–PEG. By adjusting the ratio of starting materials, different content of PEG in resulting polymers could be achieved. PAKG–8diol–PEG with higher ratio of PEG was synthesized but was readily soluble in water, resulting in difficulty in making microparticles (MPs) for our applications.

As indicated in the contact angle results (Figure S4A, Supporting Information), PAKG–8diol has higher hydrophilicity than PAKG–10diol due to the shorter aliphatic carbon chain of 1,8‐octanediol. The 1,8‐octanediol is the largest aliphatic diol that is considered water soluble while 1,10‐decanediol is water insoluble. The presence of PEG was another important factor that could influence the hydrophilicity of the polymers. The polymers with PEG had smaller contact angle which indicated higher hydrophilicity. Although PAKG–8diol showed higher hydrophilicity than PAKG–10diol, PAKG–10diol–PEG exhibited higher hydrophilicity than PAKG–8diol–PEG due to higher PEG ratio in the polymer chain. PAKG MPs were then fabricated using an oil‐in‐water emulsion method, which is illustrated in Figure 1C. By altering the energy input method, i.e., homogenization or sonication, MPs size could be controlled precisely. After fabrication, MPs sizes and morphology were characterized using SEM. The SEM images showed that all MPs fabricated using a homogenizer were spherical and had the size around 1 μm and there was no significant difference between polymers, as shown in Figure 1D.

### 2.2 Effect of PAKG MPs on Osteoblastic Differentiation and Mineralization

**Figure 2:**
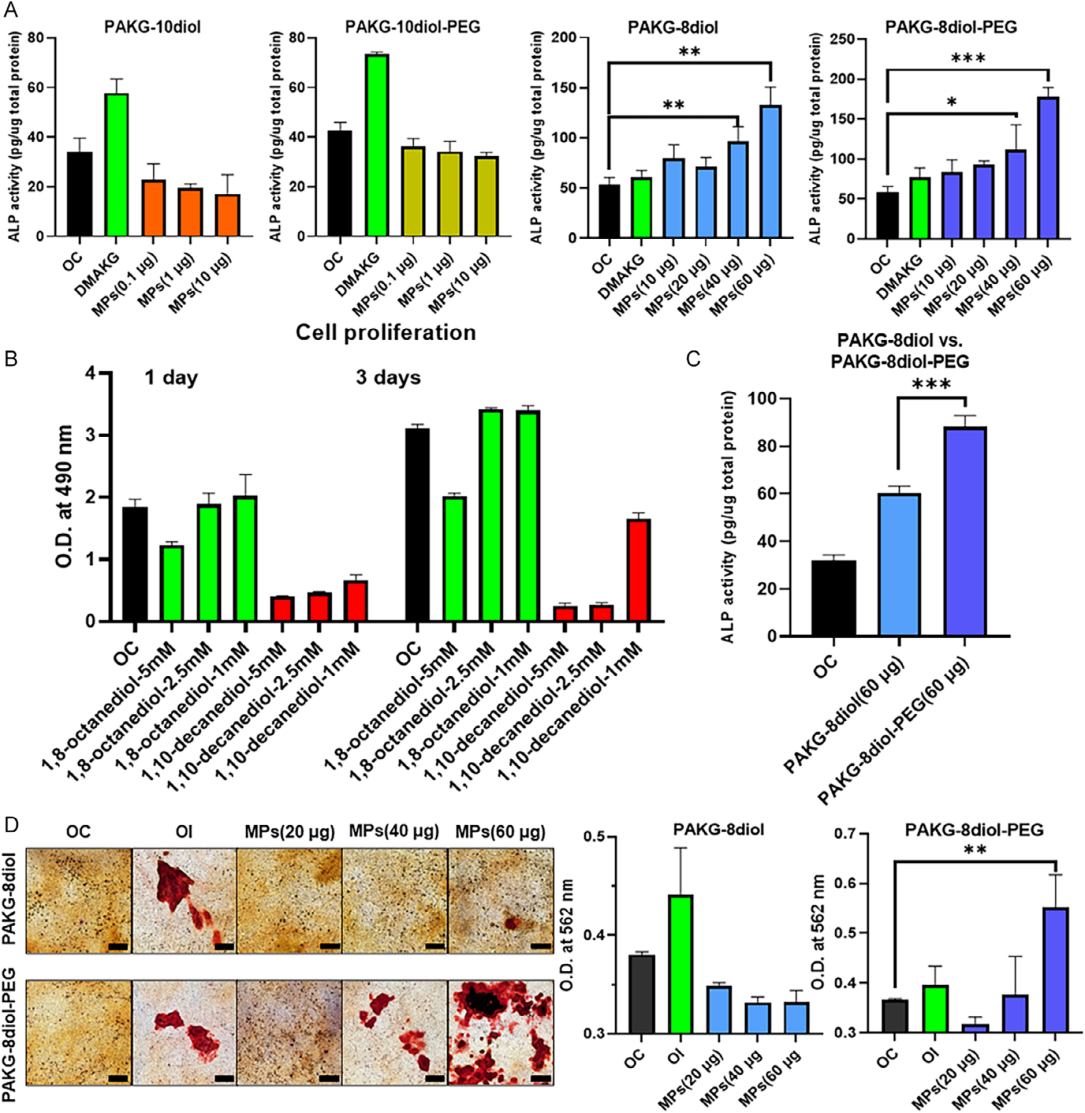
Rationale of AKG polymer design, selection (effect of carbon chain length of diol and PEG), and PAKG MPs‐enhanced osteoblastic differentiation and mineralization. A) ALP activity of MC3T3‐E1 cultured with different doses of PAKG MPs for 7 days. B) MC3T3‐E1 cell proliferation with different concentrations of 1,8‐octanediol and 1,10‐decanediol. C) Improved ALP activity of MC3T3‐E1 cells by the same dose of PAKG–8diol and PAKG–8diol–PEG MPs on day 7. D) Microscope images of mineralization of MC3T3‐E1 with PAKG–8diol and PAKG–8diol–PEG MPs for 4 weeks after Alizarin Red S staining (scale bar = 500 μm) and Alizarin Red S quantitation after staining. Data are expressed as mean ± standard deviation (SD) (*n* = 3, *p < 0.05, **p < 0.01, ***p < 0.001).

To determine the effect on osteoblast differentiation, we treated MC3T3‐E1 cells with different doses of PAKG‐10diol MPs, and DMAKG was used as a positive control as we previously reported.^[^ ^27^ ^]^ However, PAKG–10diol MPs strongly inhibited the alkaline phosphatase (ALP) activity in a dose dependent manner (**Figure** **2**A), suggesting the potential cytotoxicity of PAKG–10diol MPs. PAKG–10diol–PEG MPs showed less inhibition of ALP but failed to improve it when a PEG moiety was introduced (Figure 2A). Alternatively, the more hydrophilic 1,8‐octanediol compared to 1,10‐decanediol was tested as previously reported.^[^ ^34^ ^]^ Both PAKG–8diol and PAKG–8diol–PEG could significantly increase ALP activity in a dose‐dependent manner with up to 60 μg of MPs per well. In addition, even the lowest dose (10 μg) showed higher promotion of ALP activity compared to DMAKG (Figure 2A).

To further characterize the biocompatibility of these PAKG polymers, we studied the effects of 1,8‐octanediol and 1,10‐decanediol, which are the degradation products of PAKG, on the cell proliferation of MC3T3‐E1 cells. The result showed that 1,8‐octanediol had little negative effect on cell proliferation up to 2.5 mM while 1,10‐decanediol significantly decreased cell proliferation from 1 mM (Figure 2B). This explained the better biocompatibility of PAKGs containing 1,8‐octanediol compared to 1,10‐decanediol. It was noted that neither 1,8‐octanediol nor 1,10‐decanediol (0.5 mM) had a detectable positive effect on ALP activity in MC3T3‐E1 cells (Figure S4B, Supporting Information). This suggests that AKG is the main component contributing to the pro‐osteogenic capability in the PAKG polymers. The addition of PEG to the PAKG MPs (PAKG–8diol–PEG MPs) further improved both the ALP activity (Figure 2C) and mineralization (Figure 2D) of MC3T3‐E1 cells when both of them were treated at 60 μg per well (Figure S4C). The pro‐osteoblastic ability of PAKG–8diol and PAKG–8diol–PEG MPs was further confirmed by gene expression of multiple osteogenic markers (ALP, osteocalcin (OCN), bone sialoprotein (BSP), Runt‐related transcription factor 2 (RUNX2)) in MC3T3‐E1 (Figure S4D, Supporting Information). Based on these data, PAKG–8diol–PEG was determined to be the best formulation of the four PAKG polymers to make MPs for osteoblastic differentiation and used for the following experiments.

### 2.3 Effect of PAKG MPs Size on Osteoblastic Differentiation and Mineralization

**Figure 3:**
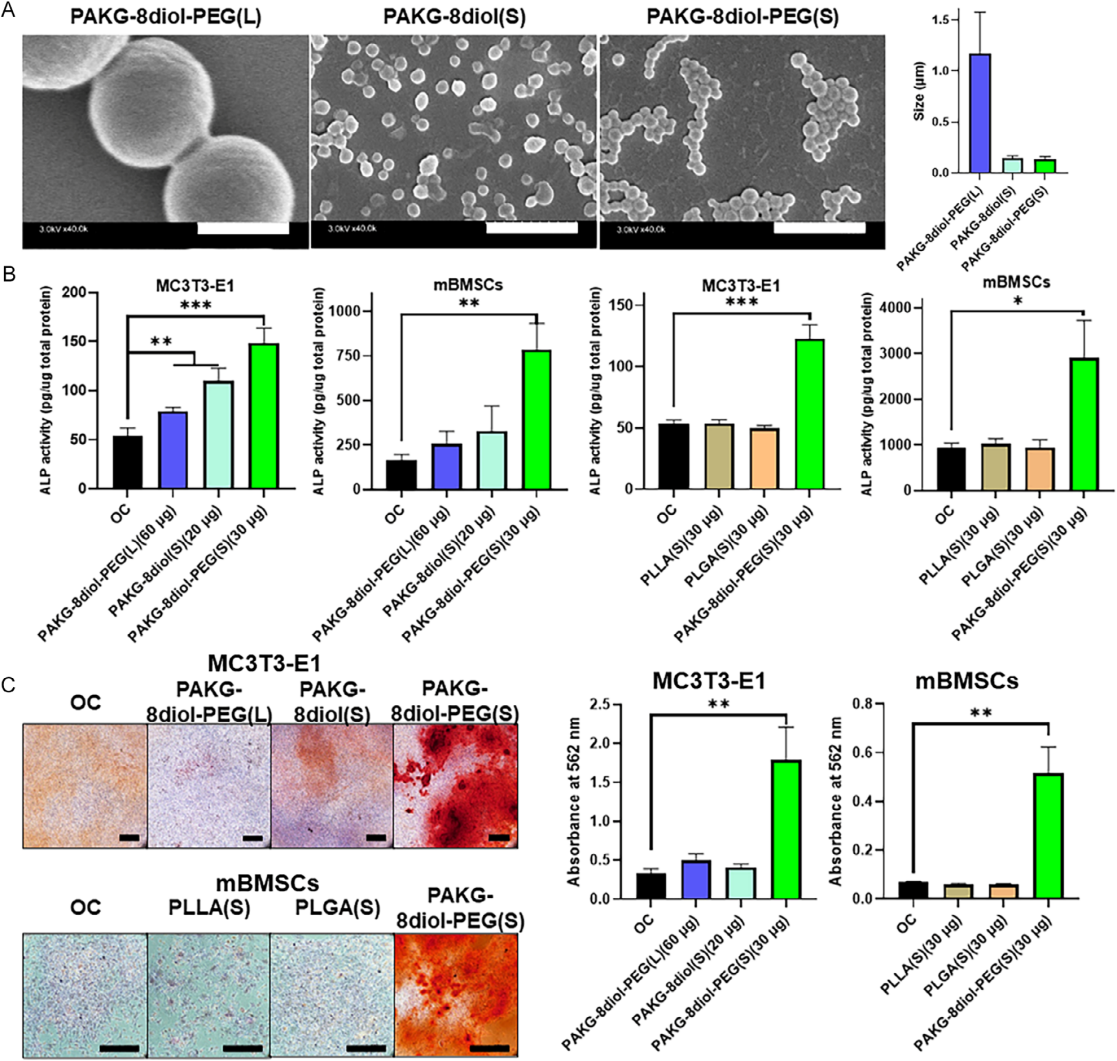
Effect of microparticle size on osteoblastic differentiation and mineralization. A) SEM images of PAKG–8diol–PEG(S), PAKG–8diol(S), and PAKG–8diol–PEG(S) MPs (scale bars = 1 μm). B) ALP activity of MC3T3‐E1 and mBMSCs with PLLA(S), PLGA(S), PAKG–8diol–PEG(L), PAKG–8diol(S), and PAKG–8diol–PEG(S) MPs. C) Microscope images of MC3T3‐E1 cultured with PAKG–8diol–PEG(L), PAKG–8diol(S), PAKG–8diol–PEG(S) MPs for 24 days and mBMSCs cultured with PLLA(S), PLGA(S), PAKG–8diol–PEG(S) MPs for 28 days after Alizarin Red S staining (left) (sale bars = 500 μm) and Alizarin Red S quantification after staining (right). Data are expressed as mean ± SD (*n* = 3, *p < 0.05, **p < 0.01, ***p < 0.001).

In addition to improving hydrophilicity, smaller size is also known to accelerate the degradation of the polymeric MPs in part due to the larger surface area. Therefore, we fabricated smaller PAKG–8diol(S) and PAKG–8diol–PEG(S) MPs to assess their bioactivities. The comparison of larger and smaller MPs is presented in **Figure** **3**A. The size of the smaller MPs was only about 1/10 of the size of the larger MPs. It was found that the optimal dose of PAKG–8diol(S) MPs was 20 μg and PAKG–8diol–PEG(S) was 30 μg (Figure S5B, Supporting Information). Then, the optimal doses of PAKG–8diol(S) and PAKG–8diol–PEG(S) were compared with the optimal dose of PAKG–8diol–PEG(L) (60 μg). The ALP activity demonstrated that smaller MPs had significantly better performance in promoting osteoblastic differentiation in both MC3T3‐E1 and primary mBMSCs (Figure 3B). The pro‐osteoblastic ability of PAKG–8diol–PEG(S) MPs was further confirmed by gene expressions of multiple osteogenic markers (OCN, BSP) (Figure S4E, Supporting Information) and mineralization (Figure 3C) using the MC3T3‐E1 cells.

For comparison, we fabricated PLLA(S) and PLGA(S) MPs with similar size to PAKG(S) MPs (Figure S5A, Supporting Information) to study their effects on the osteoblastic differentiation of MC3T3‐E1 and mBMSCs. The results indicated that neither PLLA(S) nor PLGA(S) MPs could improve the ALP activity of the tested cells. Consistently, matrix mineralization data further confirmed that small PAKG–8diol–PEG(S) MPs not the PLLA or PLGA MPs could promote osteogenic differentiation of mBMSCs (Figure 3C).

### 2.4 Osteogenesis‐Related Signaling Pathways Modulated by PAKG MPs

**Figure 4:**
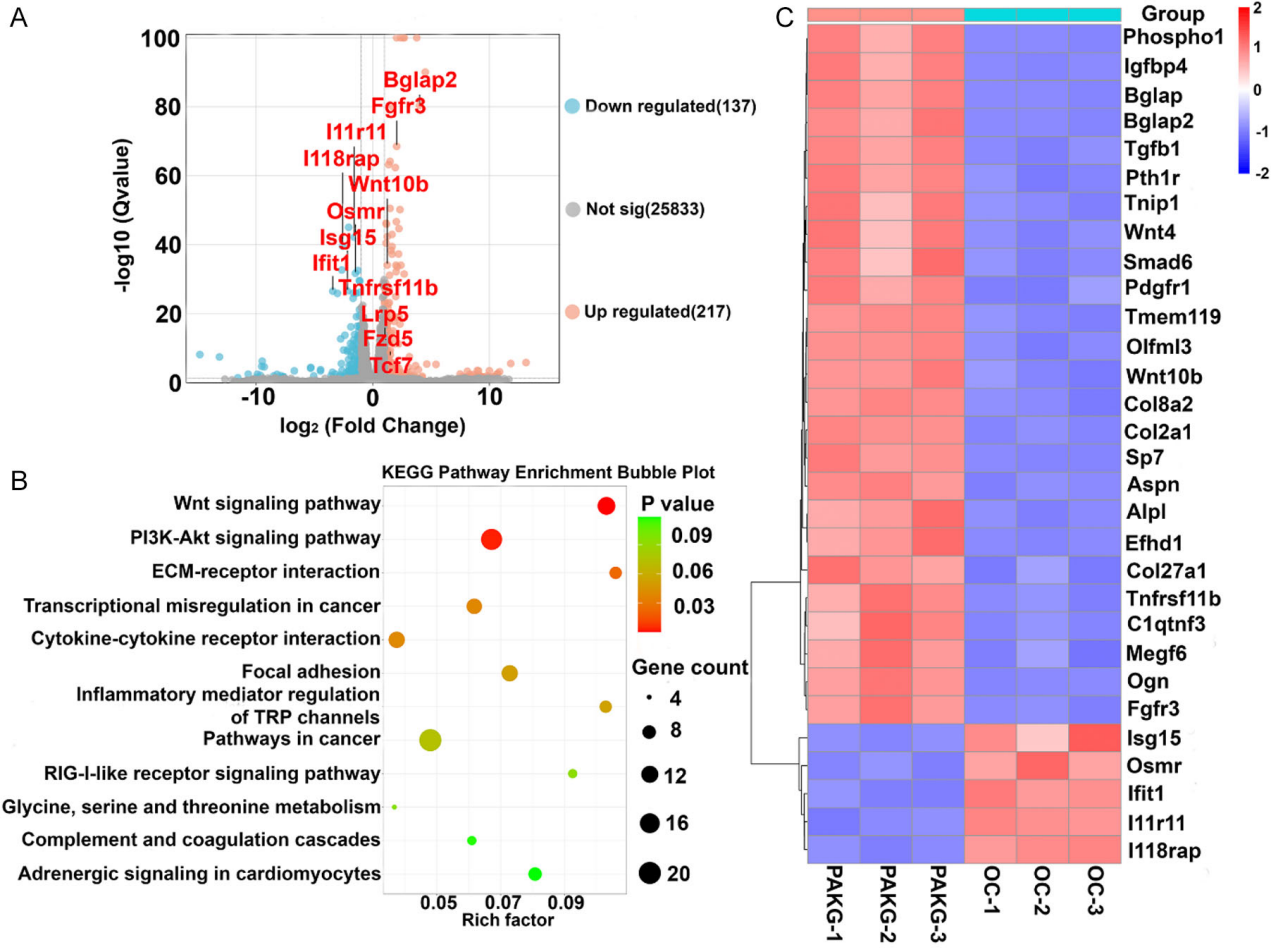
RNA‐Seq analysis of pre‐osteoblasts treated by PAKG–8diol–PEG(S) MPs (PAKG). A) Volcano plot, B) KEEG pathway enrichment bubble plot, C) heatmap of differentially expressed genes. OC: no treatment and cultured in OC medium. PAKG: PAKG MPs‐treated group also cultured in OC medium. *n* = 3.

To further characterize the pro‐osteoblastic capability of our novel PAKG–8diol–PEG(S) MPs and to elucidate the underlying mechanisms, RNA‐Seq and bioinformatics analysis using MC3T3‐E1 cells was completed. A total 217 genes were upregulated, and 137 genes were downregulated by PAKG MPs treatment compared to the nontreatment group (**Figure** **4**A). Gene ontology enrichment analysis indicated that the terms innate immune response, immune system process, ossification, regulation of bone mineralization, extracellular matrix, structural constituent of bone, and bone development were significantly upregulated in the PAKG MPs treatment group (Figure S6, Supporting Information). Furthermore, Kyoto Encyclopedia of Genes and Genomes (KEGG) enrichment analysis of differentially expressed genes revealed the top signaling pathways modulated by PAKG MPs are Wnt and PI3K–Akt signaling pathways among others (Figure 4B). More specifically, Wnt4, Wnt10b, Lrp5, Fzd5, and Tcf7 of the Wnt signaling pathway were consistently and significantly upregulated by PAKG MPs as shown in the heatmap (Figure 4C). The upregulated genes of PI3K–Akt pathways were Fgfr3, Cola2a1, and Itga10. Notably, Il1rl1, Isg15, Osmr, Ifit1, and Il18rap involved in innate immune response and inflammation were all significantly downregulated by PAKG MPs treatment. Consistent with our earlier differentiation data, the osteogenic differentiation markers, e.g., Bglap and Alpl were also significantly enhanced in the PAKG group (Figure 4C).

### 2.5 Osteoblastic Cells Internalization of PAKG MPs Mainly through Phagocytosis

**Figure 5:**
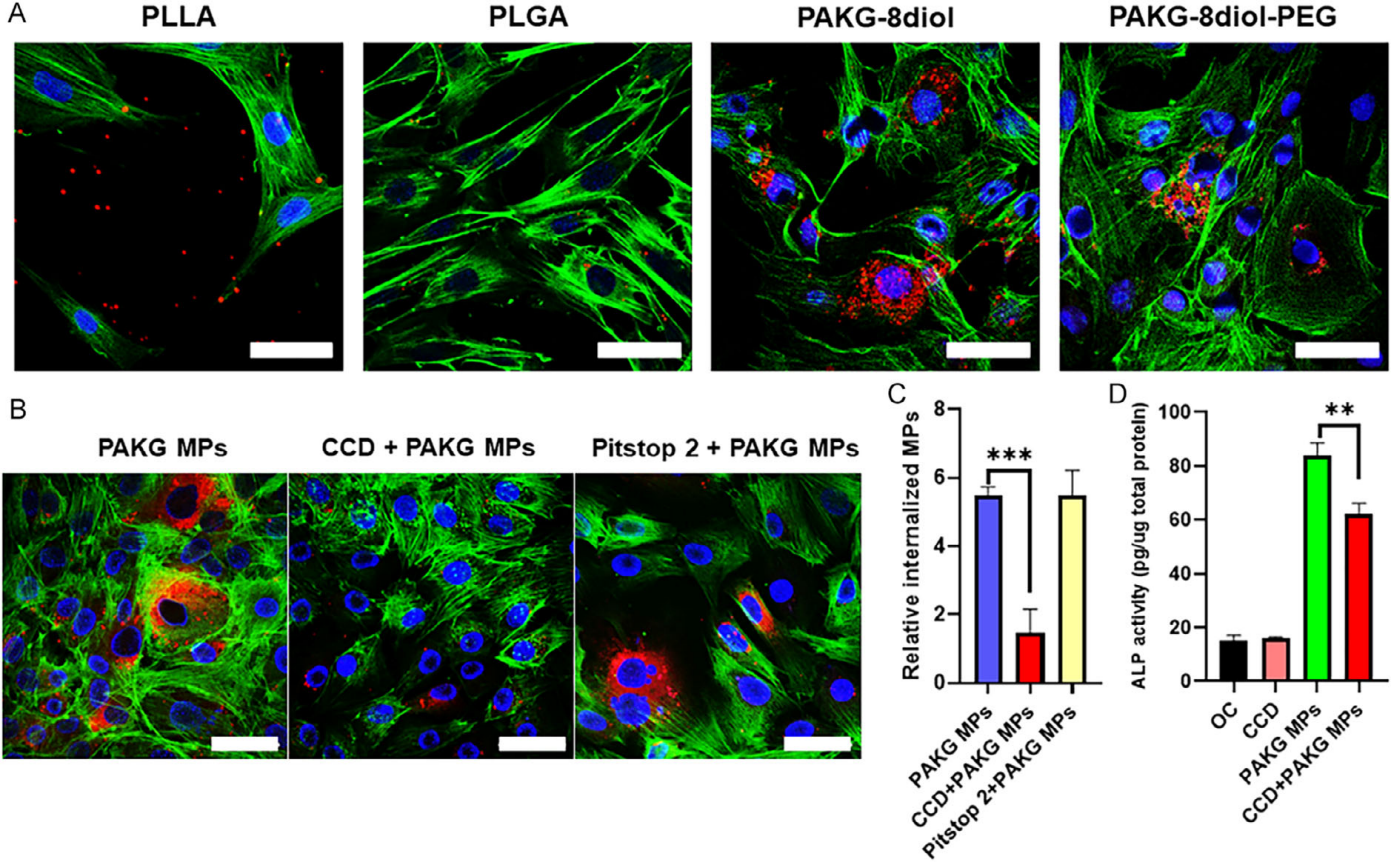
Osteoblast cell internalization of PAKG MPs through phagocytosis. A) Confocal microscopy images of 4',6‐diamidino‐2‐phenylindole (DAPI) (blue)‐ and phalloidin (green)‐stained MC3T3‐E1 cultured with different MPs. B) Confocal images of MC3T3‐E1 cultured with PAKG MPs, and PAKG MPs in the presence of cytochalasin D (CCD, a phagocytosis inhibitor, 50 nM) or Pitstop 2 (a clathrin‐independent endocytosis inhibitor, 30 μM). C) Quantitation of relative internalized MPs when treated with CCD or Pitstop 2. D) ALP activity of MC3T3‐E1 with CCD (50 nM), PAKG MPs, and PAKG MPs with CCD (50 nM). Scale bars = 50 μm. Data are expressed as mean ± SD (n = 3, **p < 0.01, ***p < 0.001).

Our data indicate that PLLA and PLGA MPs were scarcely internalized by osteoblastic cells while PAKG MPs (both PAKG–8diol and PAKG–8diol–PEG) with similar size could be easily phagocytosed (**Figure** **5**A). Cytochalasin D (CCD) is a phagocytosis inhibitor and Pitstop 2 is a clathrin‐independent endocytosis inhibitor, which were used to determine the mechanism of PAKG MP uptake by osteoblastic cells. As shown in Figure 5B,C, the data demonstrated that CCD could significantly inhibit the internalization of PAKG MPs by MC3T3‐E1 cells while Pitstop 2 had limited impact (Figure 5B,C). PAKG MPs improved ALP activity of MC3T3‐E1 cells were consistently reduced by CCD treatment (Figure 5D) suggesting that MC3T3‐E1 cells take PAKG MPs mainly through phagocytosis instead of receptor‐mediated endocytosis.

### 2.6 PAKG Enables PLGA MPs for Intracellular Drug Delivery

**Figure 6:**
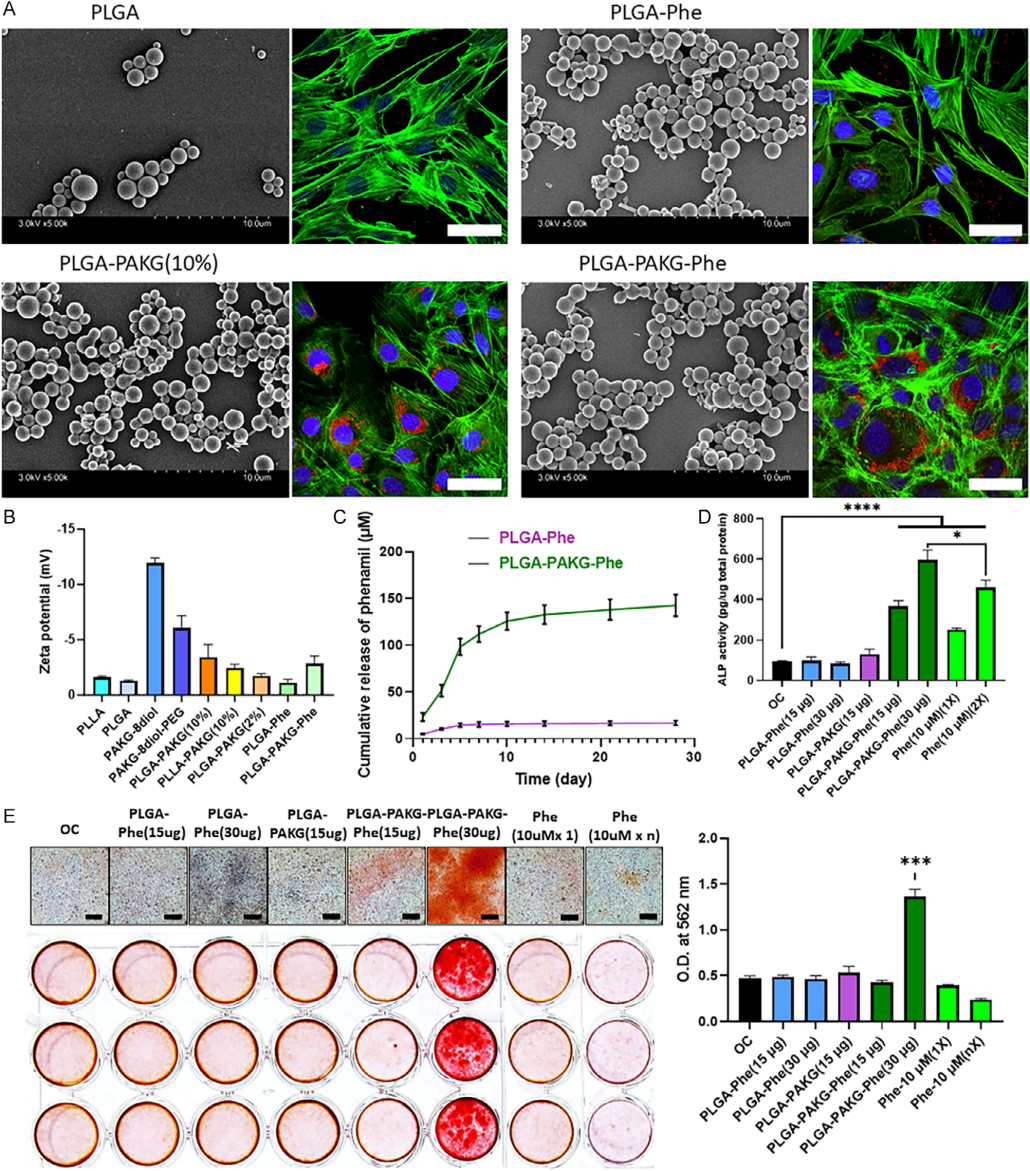
PAKG enables PLGA MPs for intracellular drug delivery. A) SEM images of different MPs (scale bars = 10 μm) and confocal microscopy images of MC3T3‐E1 cultured with MPs for overnight (scale bars = 50 μm). B) Zeta potential of various MPs. C) Release profile of phenamil from PLGA and PLGA–PAKG MPs for 4 weeks. D) ALP activity of MC3T3‐E1 on day 7. E) Alizarin Red S staining (left) and quantification after (right) MC3T3‐E1 cultured with different MPs or Phe treatment in OC medium for 2 weeks (right). Scale bars = 500 μm. Data are expressed as mean ± SD (*n* = 3, *p < 0.05, ***p < 0.001, ****p < 0.0001).

U.S. food and drug administration (FDA‐proved polymers, PLLA and PLGA, are biocompatible and biodegradable and have been widely used in drug delivery for both clinical and basic research.^[^ ^35^, ^36^, ^37^, ^38^, ^39^, ^40^, ^41^ ^]^ Our data demonstrated that PLLA or PLGA MPs alone had little uptake by non‐phagocytotic cells, e.g., osteoblasts and MSCs (Figure 5A). This lack of uptake could significantly limit their applications where intracellular drug delivery is desired. For example, small‐molecule drug, phenamil, can promote strong osteogenic differentiation by activating intracellular BMP signaling which can be facilitated by the drug carriers (e.g., MPs) with efficient internalization ability by the targeting cells (e.g., osteoblasts or stem cells). Interestingly, blending with 1% of PAKG enabled the PLGA MPs efficient phagocytosis of the PLGA–PAKG MPs (Figure S8, Supporting Information). A series of PLGA–PAKG MPs with different PAKG ratios (10, 30, 50%) were fabricated to test their effect on osteoblastic differentiation of MC3T3‐E1 cells. Interestingly, as shown in Figure S9B, Supporting Information, ALP activities of MC3T3‐E increased with the higher ratio of PAKG in the MPs. We selected 10% PAKG in PLGA and phenamil to study the effect of intracellular drug delivery of phenamil on osteoblastic differentiation of MC3T3‐E1 cells. First, we confirmed that the phagocytosis and surface property of the MPs did not change after phenamil loading. The PLGA ± Phe MPs (red) had little uptake by the cells while many PLGA–PAKG ± Phe MPs (red) were found intracellularly (**Figure** **6**A). The zeta potentials of PLGA–Phe and PLGA–PAKG–Phe MPs were close to those of PLGA and PLGA–PAKG MPs, respectively (Figure 6B). The release profile demonstrated phenamil released from PLGA–PAKG–Phe MPs was significantly faster and greater than that from PLGA–Phe MPs (Figure 6C). The subsequent ALP activity result of MC3T3‐E1 indicated that one dose of PLGA–PAKG–Phe MPs (30 μg) treatment group significantly promoted osteoblastic differentiation compared to one‐dose PLGA–Phe MPs and one‐dose or multidose free phenamil treatment groups (Figure 6D). Notably, one dose of PLGA–PAKG–Phe MPs (30 μg) accelerated the mineralization process (less than 2 weeks) compared to other treatments (Figure 6E).

### 2.7 PAKG MPs Promote Mouse Cranial Bone Regeneration

**Figure 7:**
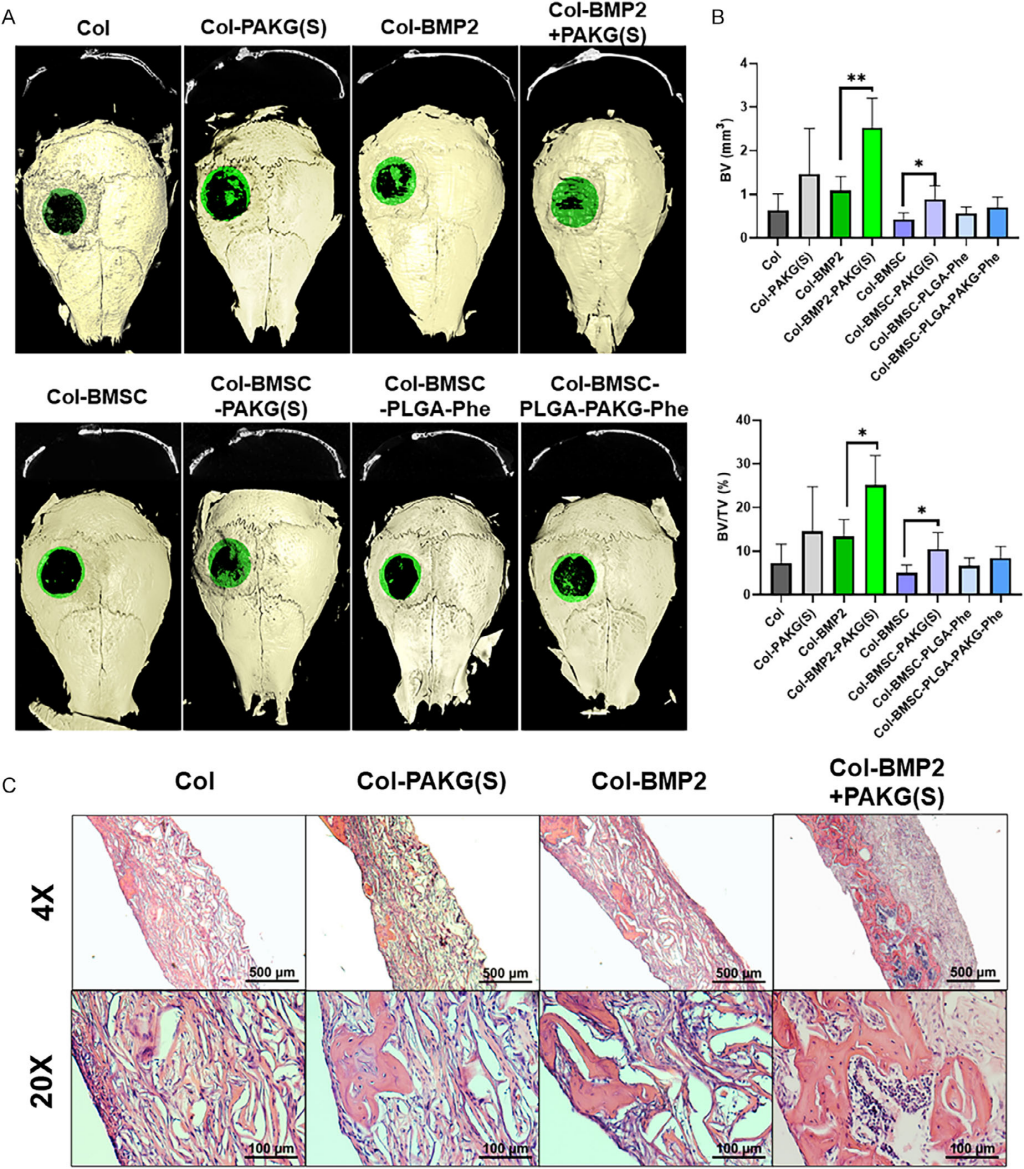
PAKG MPs promote mouse cranial bone regeneration. A) μCT top‐view and cross‐section image of mouse cranial defects after 6 weeks post‐implantation. Newly generated bone marked in green color. B) Quantification measurement of total new bone volume (BV) and the ratio of bone volume versus total volume in defected area (BV/TV) in different treatment groups. All the results were expressed as means ± SD (*n* = 3–5, *p < 0.05, **p < 0.01). C) Representative H&E‐stained tissue sections acquired from the mouse cranial defects after 6 weeks post‐implantation (scale bars = 500 μm in the upper panel and 100 μm in the lower panel).

A mouse cranial bone defect model was used to investigate if PAKG MPs can induce bone regeneration in vivo. First, we aimed to test if PAKG MPs can contribute to bone regeneration without exogenous cell transplantation. The micro‐computed tomography (μCT) data demonstrated the novel PAKG(S) MPs (Col–PAKG(S)) alone improved cranial bone regeneration compared to scaffold only (Col) (**Figure** **7**A,B). In addition, a low dose of BMP2 (0.5 μg per scaffold) had marginal bone regenerative capacity compared to the Col group. Excitingly, the combination group (Col–BMP2 + PAKG(S)) had significantly more bone regeneration compared to the Col–BMP2 group (Figure 7A,B). Second, we were interested in studying if the PAKG MPs‐treated BMSCs contributed to the new bone formation in vivo. Therefore, we used the primary mouse BMSCs isolated from the adult inbred male C57 BL6/J mice and treated with either the pro‐osteogenic PAKG(S) MPs, the PLGA–Phe or the PLGA–PAKG–Phe MPs. These BMSCs with different treatments were seeded into collagen scaffolds and then transplanted to the mouse cranial defects. As expected, the BMSCs treated with PAKG(S) MPs (Col–BMSC–PAKG(S)) had significantly more bone formation compared to the BMSCs without MPs (Col–BMSC). Surprisingly, neither the PLGA–Phe nor PLGA–PAKG–Phe could significantly improve bone formation compared to the Col–BMSC group. Moreover, BMSCs did not show any improvement for new bone formation compared to the collagen scaffold only group (Col) (Figure 7A,B). Hematoxylin and eosin (H&E) staining indicated obvious bone formation in the center of the defects from all the Col–PAKG(S), Col–BMP2, and Col–BMP2 + PAKG(S) groups while the bone formation was limited to the edge of the defects for the collagen scaffold only group (Figure 7C). Consistent with the μCT data, the histologic staining indicated that only the Col–BMSC–PAKG(S) group had bone formation in the center of the defects while other groups with BMSCs had very limited bone formation (Figure S11, Supporting Information).

## 3 Discussion

AKG is an intriguing antiaging metabolite; however, AKGs therapeutic potential for bone tissue engineering is unknown. Our PAKG–8diol and PAKG–8diol–PEG are the first AKG‐based biodegradable polymers with robust pro‐osteogenic abilities. Selection of diol and variation of PEG ratio may contribute to the biocompatibility and degradation rate.^[^ ^42^, ^43^ ^]^ Furthermore, smaller MPs have a larger surface area to weight ratio, leading to a higher degradation rate and faster AKG release.^[^ ^44^ ^]^ The cellular interaction with MPs may also change due to size differences. Therefore, both chemical and physical features of the AKG‐based polymeric MPs can be tailored for tissue regeneration and drug delivery.

Our innovative PAKG MPs are more effective at promoting both pre‐osteoblasts and primary BMSCs osteogenic differentiation and mineralization compared to the soluble AKG or DMAKG. This can be explained by the sustained release of bioactive AKG from the solid MPs for the targeted cells which is known crucial for drug efficacy.^[^ ^45^ ^]^ To better understand the pro‐osteogenic capability of the PAKG MPs, comprehensive RNA‐Seq analysis using the pre‐osteoblast cell line MC3T3‐E1 instead of heterologous primary cells, e.g., BMSCs to have better comparable information. The highly elevated osteogenic marker genes, e.g., *Alpl* (ALP) and *Bglap* (OCN) from the RNA‐Seq confirmed the pro‐osteogenic capability of PAKG MPs. Importantly, the KEGG pathway enrichment data suggests that Wnt signaling pathway is the most likely target among other pathways since several key players of Wnt pathway were significantly upregulated, e.g., *Wnt4*, *Wnt10b*, *Lrp5*, *Fzd5*, and *Tcf7*. This is not surprising because Wnt signaling pathway is well‐known for its essential anabolic roles in osteogenesis and bone regeneration.^[^ ^46^, ^47^ ^]^ Among the upregulated genes, *Wnt4* is particularly very interesting because it is produced from osteoblast and can prevent bone loss and inflammation by inhibiting nuclear factor kappa‐light‐chain‐enhancer of activated B cells (NF‐κB) in aging mice.^[^ ^48^ ^]^ This is also consistent with other immune/inflammation‐related genes, which were downregulated as our data indicated. Therefore, the osteogenic activity mediated by PAKG MPs is likely to be coupled with its anti‐inflammatory property to further promote bone regeneration. Furthermore, our data identified another top target is PI3K–Akt pathway by PAKG MPs. It is interesting to note that PI3K/AKT pathway can promote bone regeneration through its cross talk with Wnt/β‐catenin pathway.^[^ ^49^ ^]^ Wnt signaling is known to promote bone anabolism by increasing glutamine catabolism through producing AKG from the TCA cycle to activate mammalian target of rapamycin complex 1 (mTORC1) in a in a PI3K–Akt‐dependent manner.^[^ ^23^ ^]^ However, it has not been shown if increased exogenous AKG could reversely improve Wnt and PI3K/AKT signaling. Glutamine‐metabolism‐derived AKG is reported to support amino acid biosynthesis, proliferation, and osteogenic differentiation in skeletal stem cells while inhibiting their adipogenic differentiation.^[^ ^21^ ^]^ Therefore, our data suggest that PAKG MPs produced exogenous AKG which, in‐turn, promoted osteogenic differentiation through activating Wnt and PI3K/AKT signaling.

In addition to the pro‐osteogenic ability on osteoblasts, it has been reported that AKG and DMAKG can significantly modulate inflammation in macrophages.^[^ ^27^, ^50^, ^51^, ^52^, ^53^ ^]^ Our in vitro study showed that both PAKG–8diol and PAKG–8diol–PEG MPs had limited impact on LPS‐induced inflammatory cytokines production by macrophages while PAKG–10diol MPs significantly and dose‐dependently increased their expression (Figure S7, Supporting Information). In addition, previous reports demonstrate polymer nanoparticles/MPs are potent inflammatory stimulus for macrophages.^[^ ^54^, ^55^ ^]^ Additionally, the cytotoxicity of 1,10‐decanediol is significantly higher than 1,8‐octanediol as we indicated earlier. These factors might contribute to the pro‐inflammatory effect of PAKG–10diol MPs. Although AKG is also released from the gradual degradation of PAKG MPs, the concentration of AKG might be not high enough to mitigate the immense inflammatory response induced by LPS. Moreover, our PAKG MPs demonstrated strong hydrogen peroxide (H_2_O_2_)‐scavenging capacity while neither PLGA nor PLLA MPs had any functions (Figure S10A, Supporting Information). Consistently, the H_2_O_2_‐inhibited osteoblastic differentiation can be significantly rescued by PAKG MPs (Figure S10B,C, Supporting Information) because AKG is a strong antioxidant and protects cells from the detrimental ROS.^[^ ^56^, ^57^ ^]^

One intriguing feature of PAKG MPs is their ability for efficient intracellular drug delivery through phagocytosis. It is known that large particles (>0.5 μm), such as microorganisms, can be phagocytosed by phagocytic cells, i.e., macrophages, neutrophils, and DCs.^[^ ^58^, ^59^, ^60^, ^61^ ^]^ Our data indicated that the majority of PLLA(L) or PLGA(L) MPs stayed away from the cells, suggesting the repulsion between the MPs and the cells. The repulsion could be electrostatic repulsion from the surface charge of the cells and MPs (zeta potential).^[^ ^62^ ^]^ The cell membrane surface charge is usually negative.^[^ ^63^ ^]^ The zeta‐potential results showed that PAKG–8diol(L) and PAKG–8diol–PEG(L) MPs had even stronger negative charge than PLLA (L) and PLGA (G) MPs. Another possible factor is the hydrophilicity of the polymer although PAKG–8diol has similar hydrophilicity with PLLA and PLGA. Therefore, neither surface charge nor hydrophilicity can explain why PAKG MPs can be effectively phagocytosed by osteoblasts. Excitingly, our study indicates that a small portion of PAKG can enable efficient phagocytosis of PLLA or PLGA MPs for intracellular drug delivery. This ability of PAKG has many potential important applications since both PLLA and PLGA have been widely used with established safety profiles and versatile processability.^[^ ^35^, ^36^, ^40^, ^64^, ^65^ ^]^ Phenamil has shown pro‐osteogenic capabilities in recent studies.^[^ ^32^, ^33^, ^66^ ^]^ However, its application has been limited by its poor aqueous solubility^[^ ^31^ ^]^ and low bioavailability.^[^ ^32^, ^67^, ^68^ ^]^ In other words, lower concentration of phenamil has limited pro‐osteogenic capability and higher concentration causes cell death due to the high toxicity. Our results reveal that PLGA–PAKG‐MPs‐mediated intracellular delivery of phenamil significantly promoted osteoblastic differentiation and accelerated matrix mineralization, compared to PLGA‐MPs‐mediated extracellular delivery of phenamil or free phenamil. As shown in the release profile, blending 10% of a more hydrophilic PAKG in PLGA significantly accelerates degradation and thus increases the release rate of phenamil. Consequently, the phenamil is released intracellularly by PLGA–PAKG MPs in a relatively high concentration and in a sustainable way for over 10 days. In contrast, PLGA‐MPs‐mediated extracellular delivery of phenamil is too low to stimulate osteoblastic differentiation. Free phenamil at its optimal dose (10 μM from our previous studies) in the culture medium is refreshed when changing culture medium. Therefore, PLGA–PAKG–Phe group has the highest ALP activity and fastest matrix mineralization.

As expected, our in vivo data indicated that PAKG MPs significantly improved cranial bone regeneration especially when they combined with the low‐dose BMP2. The PAKG MPs alone group showed the trend to improve bone formation although there was no statistical difference compared to control group. This could be the relative low sample number (*n* = 3) due to the unexpected sample loss. Unexpectedly, transplanted BMSCs did not improve the overall bone formation compared to the groups without cells. Similarly, we did not see the improvement by the phenamil‐loaded PLGA or PLGA–PAKG MPs as in vitro studies. We used the primary BMSCs from the inbred C57BL/6J mice for our in vivo implantation studies because they were considered as autologous cells with same gene background. However, some studies reported that autologous BMSCs did not generate bone in C57BL/6 mice because the recipient T‐cells‐mediated strong inflammatory response by producing interferon gamma, tumor necrosis factor alpha, or transforming growth factor beta.^[^ ^69^, ^70^ ^]^ This might possibly explain why all the BMSCs‐transplanted group did not contribute to improved bone formation. We may use nude mice (without T cells) to study cells/PAKG‐MPs‐mediated bone regeneration in the future although our current data already confirmed PAKG MPs's strong pro‐osteogenic ability both in vitro and in vivo.

## 4 Conclusion

In summary, the novel PAKG MPs composed of AKG, diol, and PEG (PAKG–8diol–PEG) that can sustain the release of AKG upon degradation were synthesized. Our in vitro data suggests both the chemical components of the copolymers, i.e., carbon chain length of the diol and hydrophilicity, and the size of the MPs can significantly affect their cytotoxicity and pro‐osteogenic activity. Our RNA‐Seq data suggest that PAKG MPs produced exogenous AKG promotes osteogenic differentiation through mainly activating Wnt and PI3K/AKT signaling. PAKG MPs were internalized by osteoblasts mainly through phagocytosis, while PLLA or PLGA MPs with similar size could be barely internalized. Blending small ratio of PAKG with PLGA (PLGA–PAKG) could enable efficient phagocytosis for efficient intracellular delivery of drugs. PLGA–PAKG‐MPs‐mediated intracellular delivery of a model drug, phenamil, significantly promoted osteoblastic differentiation, and accelerated mineralization, compared to PLGA‐MPs‐mediated extracellular delivery of phenamil or free phenamil. Consistent with the in vitro data, our in vivo data confirmed that PAKG MPs significantly improved the large bone regeneration in a mouse cranial bone defect model. Thus, the novel PAKG‐based MPs showed great promise to improve osteogenic differentiation and bone regeneration and enable efficient intracellular drug delivery through phagocytosis for broad regenerative medicine.

## 5 Experimental Section

5.1

5.1.1

##### Materials

α‐AKG, 1,10‐decanediol, 1,8‐octanediol, poly (ethylene glycol) (6000) were purchased from Sigma (St. Louis MO, USA). Phenamil was purchased from Cayman Chemical (Ann Arbor MI, USA). Poly(L‐lactide) ester terminated (1.06 dL g^−1^) and 50:50 poly (DL‐lactide*‐co*‐glycolide) (0.39 dL g^−1^) were purchased from DURECT Corporation (Birmingham AL, USA). Other chemical reagents were of analytical grade. The homogenizer was IKA dispenser (T 25 digital ULTRA TURRAX) with an 18G tip. Sonicator was Branson Sonifier 450 with a 1/4″ tip.

##### Synthesis of PAKG–10diol and PAKG–8diol

An amount of 1 g of α‐AKG was mixed with 1.2 g of 1,10‐decanediol or 1 g of 1,8‐octanediol in a round‐bottom flask without solvent. The round‐bottom flask was connected to vacuum and heated to 120 °C in an oil bath. The solid mixture melted upon heating and kept magnetic stirring for 36 h. After reaction, the resulting polymer was dissolved in minimal amount of dichloromethane and precipitated in methanol. The precipitated polymer was collected by centrifuge at 1000 rpm in a conical tube and washed with methanol 3 times to remove unreacted monomers. Finally, the polymers were dried in vacuum desiccator. The polymers were characterized by 1H NMR spectroscopy and the molecular weight was evaluated with gel permeation chromatography.

##### Synthesis of PAKG–10diol–PEG and PAKG–8diol–PEG

An amount of 1 g of α‐AKG and 1 g of PEG (6 K) were mixed with 1.2 g of 1,10‐decanediol or 1 g of 1,8‐octanediol in a round‐bottom flask without solvent. The round‐bottom flask was connected to vacuum and heated to 120 °C in an oil bath. The solid mixture melted upon heating and kept magnetic stirring for 36 h. After reaction, the resulting polymer was dissolved in minimal amount of methanol and precipitated in water. The precipitated polymer was collected by centrifuge at 1000 rpm in a conical tube and washed with water 3 times to remove unreacted monomers. Finally, the polymers were dried in vacuum desiccator. The polymers were characterized with 1H NMR spectroscopy and PEG content in polymers was determined based on 1H NMR spectrum and the molecular weight was evaluated with gel permeation chromatography.

##### Contact Angle Measurement

To evaluate hydrophobicity/hydrophilicity of PAKG–10diol, PAKG–10diol–PEG, PAKG–8diol, and PAKG–8diol–PEG, water contact angle measurement was conducted with contact angle goniometer. In brief, 10 mg of polymer was dissolved in 0.2 mL of dichloromethane. One a glass slide, polymer solution was spread evenly with pipette and let solvent to evaporate. A thin film of polymer formed on the glass slide. Then, a water droplet was injected onto the polymer film and an image of the droplet was captured.

##### Large MPs Fabrication and Characterization

PAKG(L) MPs were fabricated using an oil‐in‐water emulsification and solvent evaporation method. In brief, 50 mg of the PAKG polymers were dissolved in 1 mL of dichloromethane. The polymer solution was added to 10 mL of polyvinyl alcohol (PVA) (2%) in deionized water under homogenization at 10 000 rpm. After 30 s homogenization, the emulsion was poured into 50 mL of PVA (1%) solution and stirred at 300 rpm overnight to ensure complete removal of dichloromethane. The MPs were collected by centrifuge at 1000 g in a conical tube and then washed with deionized water five times to ensure complete removal of PVA. The MPs were finally resuspended in deionized water and the concentrations of MPs were determined by freeze drying a specific volume of suspension and weighing the MPs. The size and morphology of the MPs were analyzed with SEM. Zeta potential of MPs was analyzed with zeta sizer.

##### Small MPs Fabrication and Characterization

PAKG(S) MPs were also fabricated using an oil‐in‐water emulsification and solvent evaporation method. In brief, 50 mg of the PAKG polymers were dissolved in 1 mL of dichloromethane. The polymer solution was added to 10 mL of PVA (2%) solution under probe sonication. After 2 min of sonication, the emulsion was poured into 50 mL of PVA (1%) solution and stirred at 300 rpm overnight to ensure complete removal of dichloromethane. The MPs were collected by ultracentrifuge at 16000 g in a ultracentrifuge tube and then washed with deionized water five times to ensure complete removal of PVA. The MPs were finally resuspended in deionized water and the concentrations of MPs were determined by freeze drying a specific volume of suspension and weighing the MPs. The size and morphology of the MPs were analyzed with SEM.

##### Phenamil‐Encapsulated MPs Fabrication

Phenamil‐encapsulated MPs were fabricated with PLGA (PLGA–Phe) or 90% of PLGA and 10% of PAKG–8diol–PEG (PLGA–PAKG–Phe) using the same method mentioned earlier. In brief, 50 mg of PLGA or 45 mg of PLGA and 5 mg of PAKG–8diol–PEG were dissolved in 1.8 mL of DCM. Then, 5 mg of phenamil in 200 μL of DMSO was added to the polymer solution. The mixture solution was added to 10 mL of PVA (2%) in deionized water under homogenization at 10 000 rpm. After 30 s homogenization, the emulsion was poured into 50 mL of PVA (1%) solution and stirred at 300 rpm overnight to ensure complete removal of dichloromethane. The MPs were collected by centrifuging in a conical tube and then washed with deionized water five times to ensure complete removal of PVA. The MPs were finally resuspended in deionized water and the concentrations of MPs were determined by freeze drying a specific volume of suspension and weighing the MPs.

##### Phenamil Release Study

Phenamil release from PLGA–Phe and PLGA–PAKG–Phe MPs were studied using ultraviolet–visible (UV–vis) spectroscopy. First, a standard curve of phenamil in phosphate‐buffered saline (PBS) buffer was generated by preparation of phenamil in different concentrations (0, 1.25, 2.5, 5. 10, 20 μM). Then, 4 mg of PLGA–Phe and PLGA–PAKG–Phe MPs were measured in a 1.5 mL centrifuge tube, respectively, and added 1 mL of PBS buffer for release test. Each sample was performed in triplicate. At each time point, the sample was centrifuged at 5000 rpm for 10 min and the supernatant was collected for UV–vis spectroscopy test. Then, 1 mL of fresh PBS was added to the sample tube for continuing phenamil release. The release was conducted for a total time of 4 weeks.

##### Antioxidation Property

AKG was reported to be capable of scavenging‐reactive oxygen species. To test the scavenging capability of PAKG–8diol–PEG MPs, we incubated 2 mg of PAKG–8diol–PEG MPs with hydrogen peroxide (100 μM). In comparison, we used 1 mg of AKG (free acid form), Na_2_AKG (disodium salt form) and DMAKG to incubate with hydrogen peroxide. Negative control groups were hydrogen peroxide with addition of PLLA and PLGA MPs. After 30 min of incubation, hydrogen peroxide concentration in each group was measured with hydrogen peroxide detection kit.

##### Cell Culture and Cell Seeding

Mouse‐calvaria‐derived pre‐osteoblasts (MC3T3‐E1, from ATCC), mouse BMSCs isolated from adult male C57BL/6J mice as we previously described,^[^ ^71^ ^]^ human bone marrow mesenchymal stem cells (hMSCs, purchased from Lonza) were used in the in vitro cell experiments. These cells were cultured in growth medium (GM) in 10 cm polystyrene cell‐culture‐treated dish under a humidified atmosphere with 5% CO_2_ at 37 °C. GM was prepared with minimum essential medium α (1X) (Gibco, Waltham, MA), supplemented with 10% fetal bovine serum, 100 μg mL^−1^ streptomycin sulfate, and 100 U mL^−1^ penicillin. For in vitro cell experiments, 10 000 cells (MC3T3‐E1, BMSCs, or hMSCs) in 0.5 mL of GM were seeded in 24 well plate with triplicate in each experimental group. Cells were allowed to attach and proliferate for 2 days before drug/MPs treatment.

##### Cell Viability and Proliferation

Cytotoxicity of 1,8‐octanediol and 1,10‐decanediol on MC3T3‐E1 cells were evaluated using a CellTiter 96 Aqueous One Solution Cell Proliferation Assay (MTS) according to the product instructions (Promega, Madison, WI, USA) on day 1 and 3. Reacted medium was measured at 490 nm using a SpectraMax M2e Microplate Reader (Molecular Devices, San Jose, CA, USA).

##### MPs Treatment of Cells

For each group of cells on 24‐well plate, a certain amount of MPs in a 2 mL centrifuge tube was added 1.5 mL of osteoconductive medium (OC medium). OC medium was prepared with GM supplemented with 50 μg mL^−1^ of L‐ascorbic acid and 2.5 mM of β‐glycerophosphate salt. The MPs suspension was sonicated in bath sonicator for 5 min to minimize MPs aggregation. After which the GM in each well was replaced with the suspension. Culture medium was refreshed with OC medium every 3–4 days without adding additional MPs.

##### Gene Expression and RNA‐Seq Analysis

Quantitative gene expression analysis was conducted following established protocols. In brief, RNA was extracted and purified using the miRNeasy Mini kit (Qiagen) following the manufacturer's protocol. Subsequently, the isolated RNA served as the template for cDNA synthesis, achieved through the iScript cDNA Synthesis Kit (Bio‐Rad). Then, the quantitative polymerase chain reaction (PCR) was executed using iQ SYBR Green Supermix (Bio‐Rad) and evaluated using a Bio‐Rad C1000 Touch PCR thermal cycler. The osteogenic biomarkers, including OCN, ALP, BSP, and RUNX2, were employed for assessing osteoblastic differentiation in MC3T3‐E1, with gene expression analysis conducted after 7 days of culture. Gene primers of OCN, ALP, BSP, and RUNX2 were purchased from Bio‐Rad Laboratory, Inc. while the housekeeping gene primer glyceraldehyde 3‐phosphate dehydrogenase was prepared at the University of Iowa.

For RNA‐Seq study, mouse MC3T3‐E1 cells were treated with PAKG–8diol–PEG(S) MPs (30 μg mL^−1^) for 2 days before cell harvested for total RNAs isolation. The cells without MPs treatment and cultured in the same OC medium were served as control group. Poly(A) RNA‐sequencing library construction and sequencing, transcripts assembly, and different expression analysis were performed by LC Sciences (Houston, TX). R package DESeq2 was used to perform the mRNAs differential expression analysis between the two groups. The mRNAs with the parameter of false discovery rate (specifically *Q*‐value) below 0.05 and absolute fold change ≥2 were considered differentially expressed mRNAs.

##### Confocal Microscopy

MC3T3‐E1 cells were used for confocal microscopy to visualize phagocytosis of MPs. In brief, MC3T3‐E1 cells were seeded on a round glass cover slip with 15 mm in diameter which could fit in the well of 24‐well plate. After cell adhesion, MPs labeled with Rhodamine B were added. After 24 h, cells were fixed with 10% formaldehyde for 1 h and then washed 2–3 times with PBS to remove formaldehyde. A 0.1% Triton X‐100 in PBS was added to the fixed cells for 5 min to increase permeability and washed cells 23 times with PBS. The actin structure was stained with fluorescent phalloidin and nuclear was stained with DAPI. Then, the cells with MPs were visualized with confocal microscopy.

##### Animal Study

Laboratory animals’ care and use were followed by the protocols approved by the office of the Institutional Animal Care and Use Committee (IACUC) of the University of Iowa. The IACUC Administrator, Gwen Waddingham, approved the animal protocol with the number 4 062 153. Inbred C57BL/6J male mice (8–9 weeks, JACKSON LABS) were used to create the large size cranial bone defect model for in vivo bone regeneration study as we previously reported.^[^ ^72^, ^73^ ^]^ Briefly, one 3 mm bone defect was created within the parietal bone using a trephine bur while the underlying dura mater was kept intact. The sterile collagen foam/MPs/cells/BMP2 were directly placed on the cranial defects and the overlying tissue was closed with surgical staples. Staples were removed 7 days after surgery. The porous resorbable collagen foam (ACE Surgical Supply Co., Inc.) was cut into 4 × 4 × 1.5 mm cuboids. These collagen scaffolds were further cross‐linked using N‐(3‐dimethylaminopropyl)‐N’‐ethylcarbodiimide hydrochloride^[^ ^72^ ^]^ for 2 h and functionalized with hydroxyapatite as previously described^[^ ^74^ ^]^ to improve their mechanical properties and osteo‐conductivity, respectively. A total of 40 mice were used in our animal experiments, divided into 8 groups (*n* = 5) and treated as follows: 1) Col group: modified collagen scaffolds were implanted to the defects as the scaffold‐only control group. 2) Col–PAKG(S) group: collagen scaffolds loaded with 60 μg of PAKG(S) MPs were implanted to the mice. 3) Col–BMP2 group: the mice were implanted with collagen scaffolds loaded with 0.5 μg of BMP2 (rhBMP2, Peprotech, Rocky Hill, NJ, USA). 4) Col–BMP2 + PAKG(S) group: the mice were implanted with collagen scaffolds loaded with 0.5 μg of BMP2 and 60 μg of PAKG(S) MPs. 5) Col–BMSC group: the sterile collagen scaffolds were loaded with two million of primary mouse BMSCs (isolated from adult male C57BL/6J mice) and cultured in OC medium for 5 days before transplantation into the mice. 6) Col–BMSC–PAKG(S) group: the collagen scaffolds were loaded with the same number of BMSCs that treated with 30 μg mL^−1^ of PAKG(S) MPs overnight and cultured in OC medium for 5 days before transplantation into the mice. 7) Col–BMSC–PLGA–Phe group: the same number of BMSCs were treated with 60 μg mL^−1^ of PLGA–Phe MPs overnight and seeded on the collagen scaffolds then cultured in OC medium for 5 days before transplantation into the mice. 8) Col–BMSC–PLGA–PAKG–Phe group: same treatment as group (7) except using PLGA–PAKG–Phe MPs instead of PLGA–Phe MPs. The samples with cranial bones were retrieved and fixed for μCT (SkyScan 1272, Bruker) and histological analysis after all the mice were euthanized at 6 weeks after surgery. Some samples were not counted for data analysis because some animals died during or after the surgery or some implants shifted out of the defects. The counted sample number for each group (from 1 to 8 group) was 5, 3, 4, 4, 5, 5, 4, and 4, respectively.

##### Statistical Analysis

All experiments were performed with triplicates in each group, unless otherwise stated. Each experiment was repeated at least once. To determine the statistical significance of the differences between groups, a two‐sided *t‐*test using GraphPad Prism 9 was utilized to analyze the means between the control group and each experimental group. *P < 0.05; **P < 0.01; ***P < 0.001; and ****P < 0.0001 were used to identify the statistically significance between individual groups.

## Conflict of Interest

The authors declare no conflict of interest.

## Author Contributions

**Zhuozhi Wang**: Data curation (lead); Investigation (lead); Validation (lead); Writing—original draft (lead). **Jue Hu**: Investigation (supporting). **Jeffrey S. Marschall**: Writing—review and editing (supporting). **Ling Yang**: Writing—review and editing (supporting). **Erliang Zeng**: Writing—review and editing (supporting). **Shaoping Zhang**: Writing—review and editing (supporting). **Hongli Sun**: Conceptualization (lead); Data curation (supporting); Formal analysis (supporting); Funding acquisition (lead); Investigation (lead); Methodology (supporting); Project administration (lead); Resources (lead); Supervision (lead); Writing—original draft (equal); Writing—review and editing (lead).

## Supporting information

Supplementary Material

## Data Availability

The data that support the findings of this study are available from the corresponding author upon reasonable request.
