# Supplementary material for: Antiaging Metabolite‐Based Polymeric Microparticles for Intracellular Drug Delivery and Bone Regeneration
Source: Small Sci. 2024 Sep 3;4(10):2400201. doi: 10.1002/smsc.202400201 (PMC11460827; doi:10.1002/smsc.202400201)
Supplement: Supplementary file 1 — Supplementary Material [file SMSC-4-2400201-s001.pdf]

Supporting Information

Anti-aging Metabolite-Based Polymeric Microparticles for Intracellular Drug Delivery and Bone Regeneration

Zhuozhi Wang [a], Jue Hu [a], Jeffrey S. Marschall [b], Ling Yang [c], Erliang Zeng [a, d], Shaoping Zhang [a, e], Hongli Sun [a, b, f*]

[a] Iowa Institute for Oral Health Research, University of Iowa College of Dentistry, Iowa City, IA 52242, USA

[b] Department of Oral and Maxillofacial Surgery, University of Iowa College of Dentistry, Iowa City, IA 52242, USA

[c] Department of Anatomy and Cell Biology, Fraternal Order of Eagles Diabetes Research Center, Pappajohn Biomedical Institute, University of Iowa Carver College of Medicine, Iowa City, IA, USA.

[d] Division of Biostatistics and Computational Biology, University of Iowa College of Dentistry, Iowa City, IA 52242, USA.

[e] Department of Periodontics, University of Iowa College of Dentistry, Iowa City, IA 52242, USA.

[f] Roy J. Carver Department of Biomedical Engineering, University of Iowa College of Engineering, Iowa City, IA 52242, USA.

Corresponding Authors

* Professor Hongli Sun, Ph.D.

Department of Oral and Maxillofacial Surgery,

Iowa Institute for Oral Health Research,

N409 DSB, 801 Newton Road, The University of Iowa, Iowa City, IA 52242

Tel: 319-335-1217 E-mail: hongli-sun@uiowa.edu

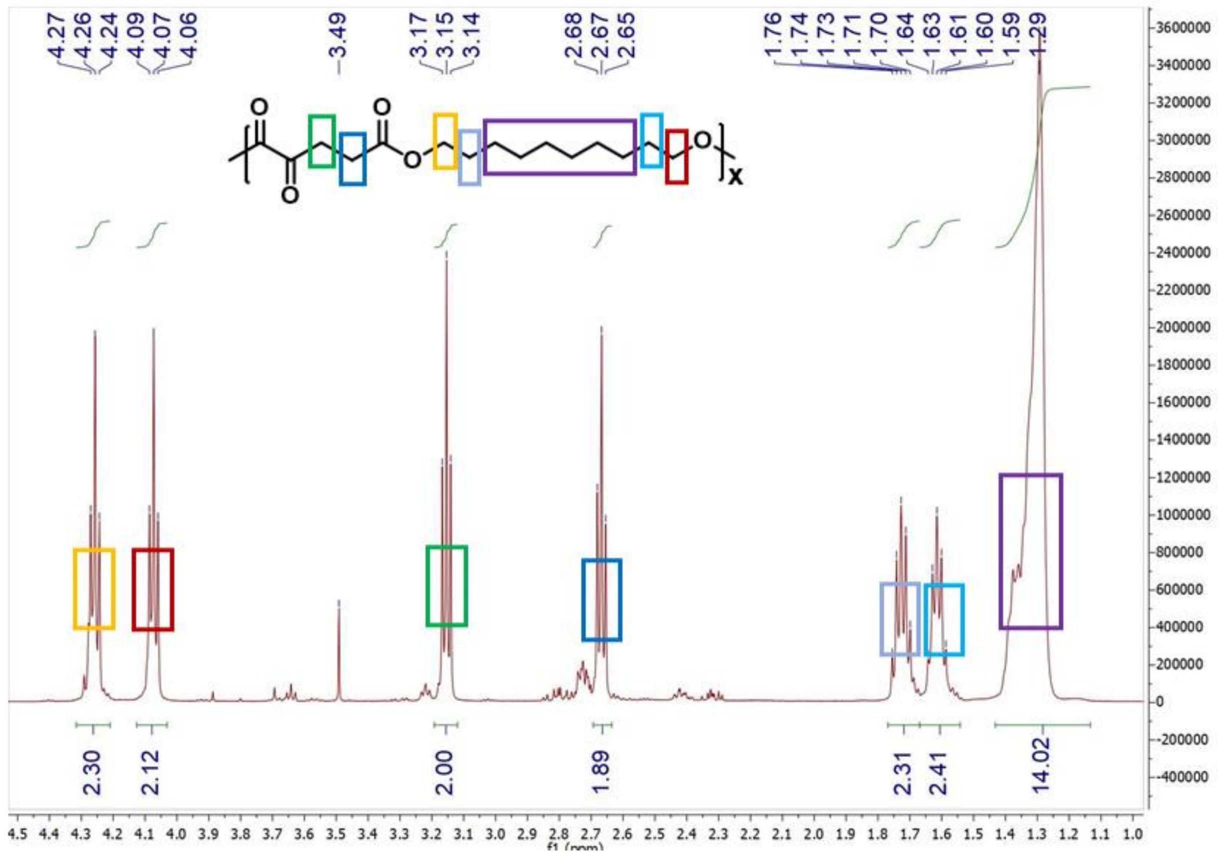

Figure S1. ^{1}H **NMR spectrum of PAKG-10diol.**

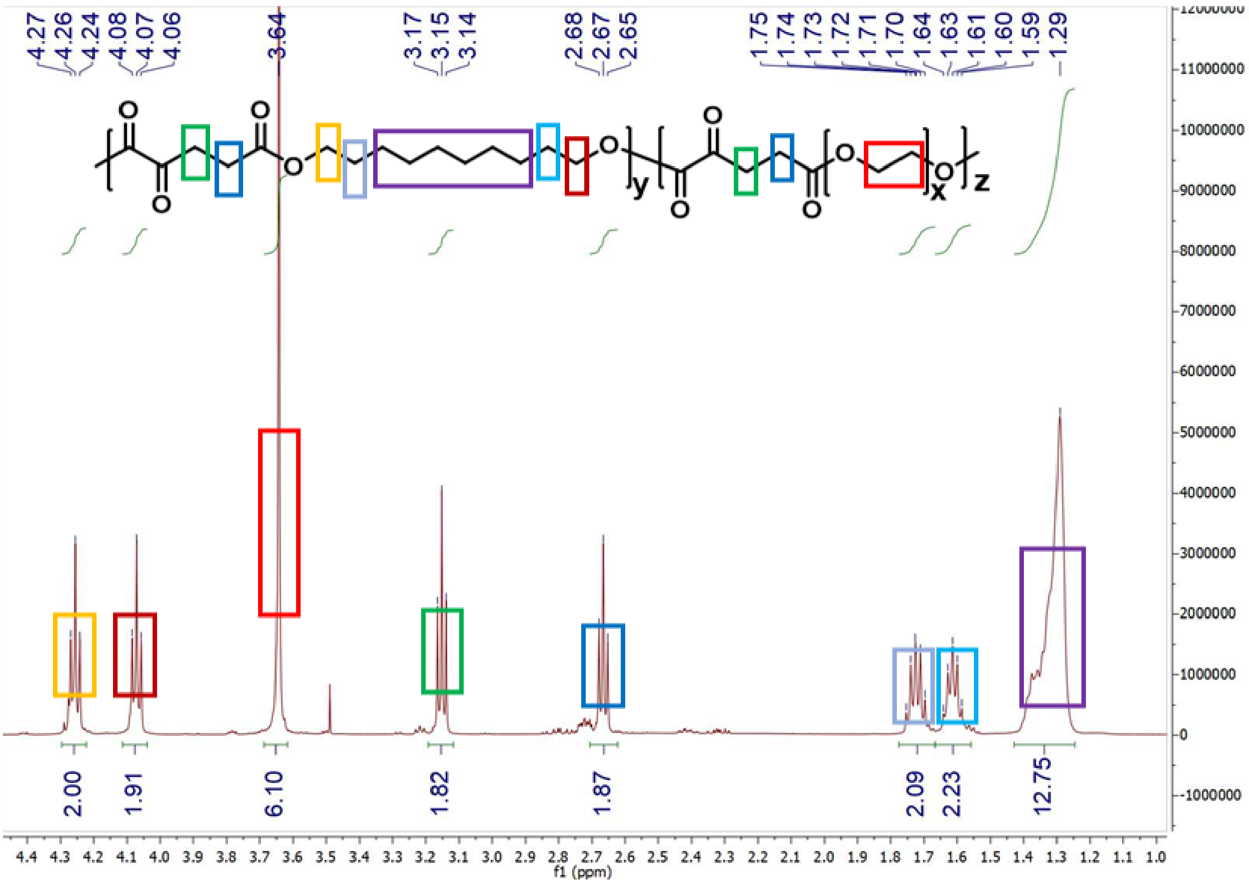

Figure S2. ^{1}**H NMR spectrum of PAKG-10diol-PEG.**

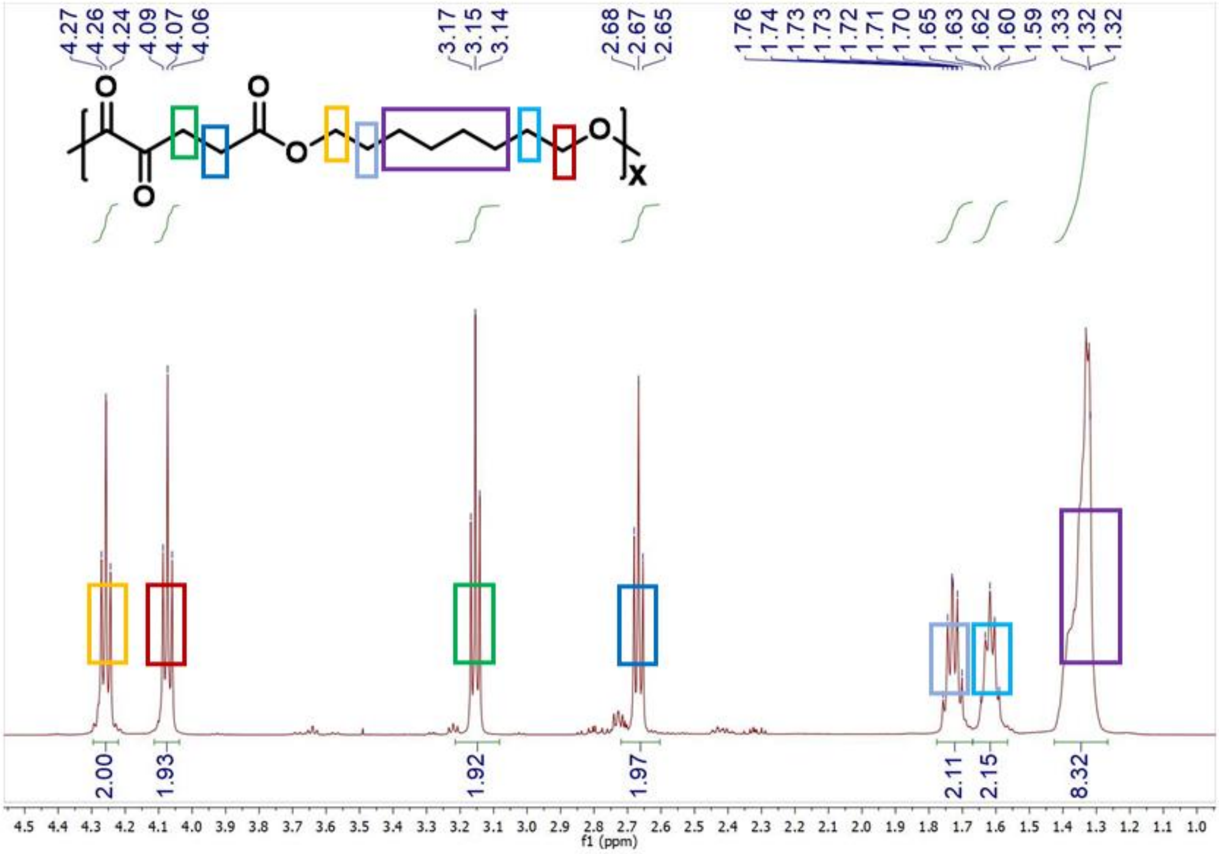

Figure S3. 1H NMR spectrum of PAKG-8diol.

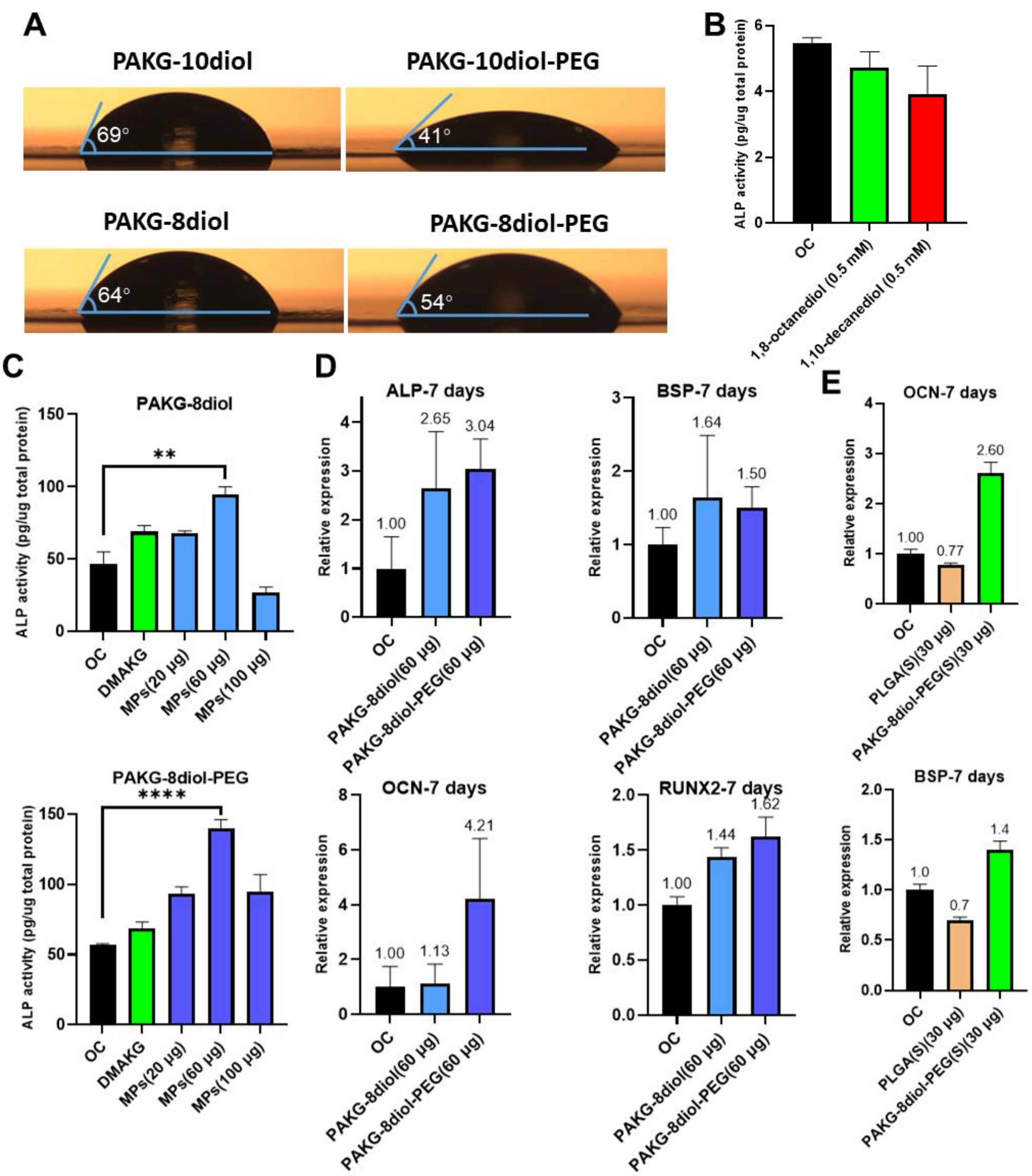

Figure S4. (A) Water contact angle on PAKG film. (B) ALP activity of MC3T3-E1 treated with same dose of 1,8-octanediol and 1,10-decanediol. MPs. (C) ALP activity of MC3T3-E1 with different dose of PAKG-8diol and PAKG-8diol-PEG. (D) Osteogenic marker gene expression (ALP, BSP, OCN, RUNX2) of MC3T3-E1 after culture with PAKG-8diol and PAKG-8diol-PEG MPs for 7 days. (E) Osteogenic marker gene expression (BSP, OCN) of MC3T3-E1 after culture with PLGA(S) and PAKG-8diol-PEG(S) MPs for 7 days. Data are expressed as mean ± SD (n = 3, *p < 0.05, **p < 0.01, ***p < 0.001, ****p < 0.0001).

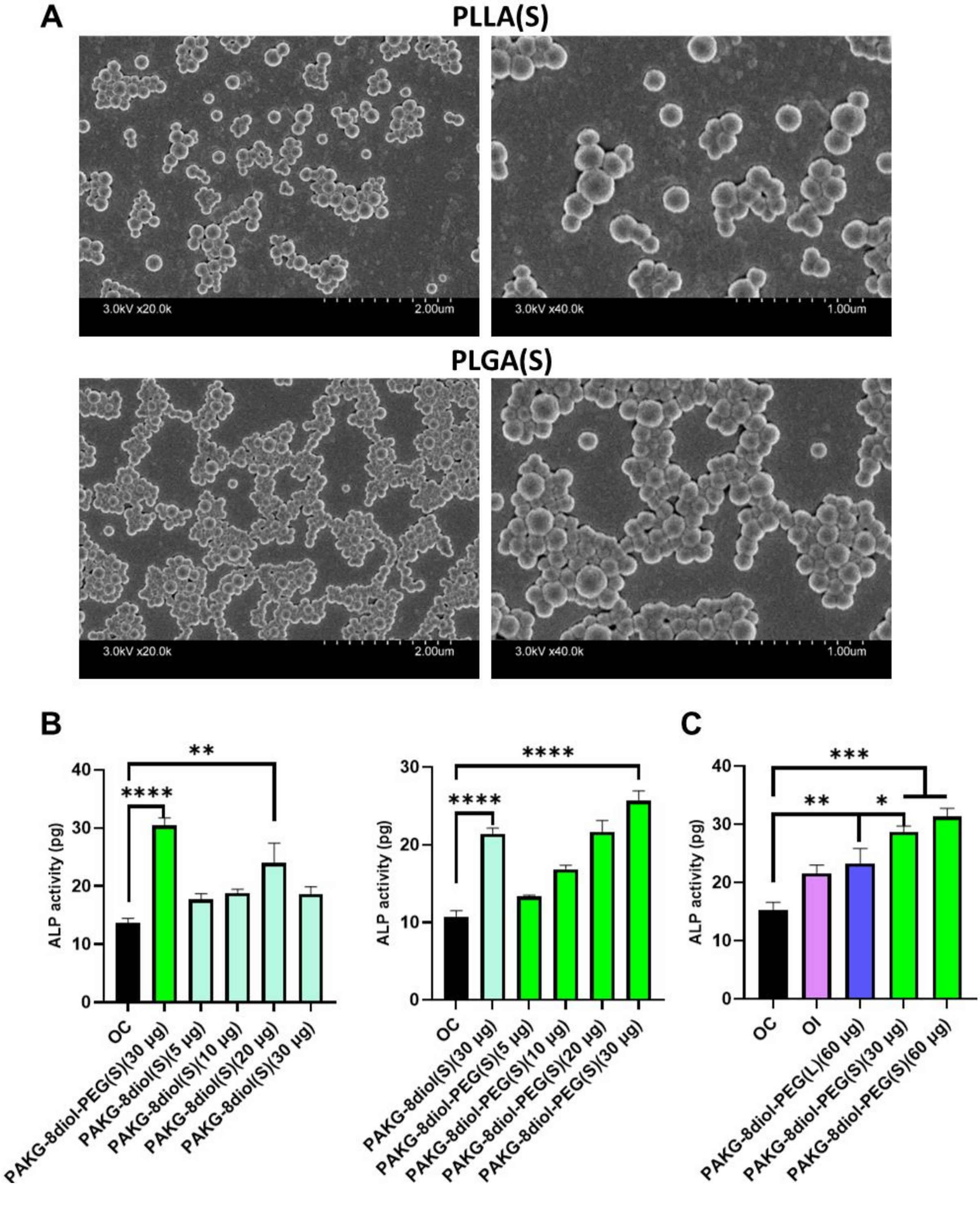

Figure S5. (A) SEM images of PLLA(S) and PLGA(S) MPs. (B) ALP activity of MC3T3-E1 with different doses of small PAKG-8diol(S) and PAKG-8diol-PEG(S) MPs for dose optimization. (C) Comparison of large PAKG-8diol-PEG(L) and small PAKG-8diol(S) and PAKG-8diol-PEG(S) MPs by ALP activity. Data are expressed as mean ± SD (n = 3, *p < 0.05, **p < 0.01, ***p < 0.001, ****p < 0.0001).

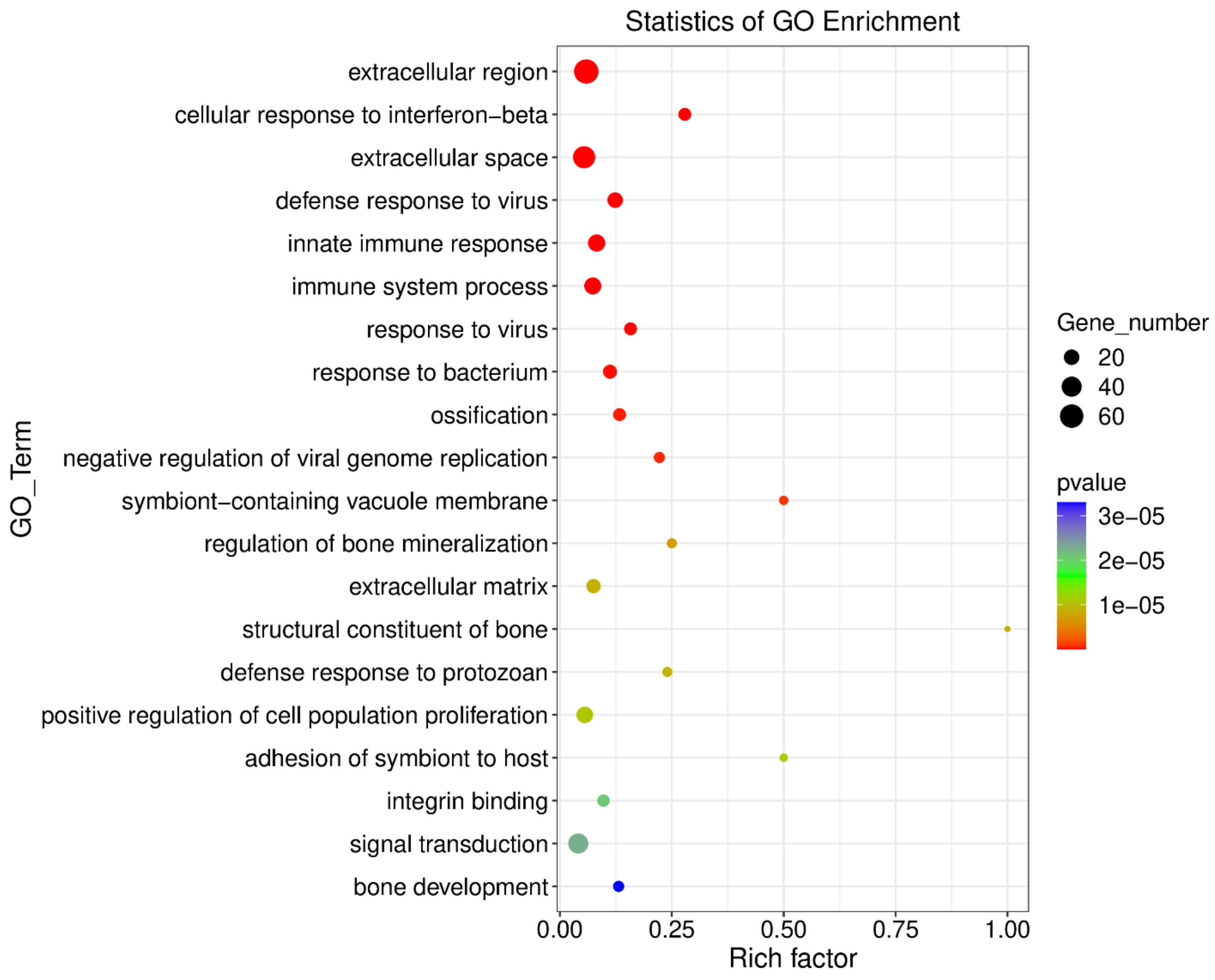

Figure S6. GO enrichment scatterplot (Treatment vs Control)

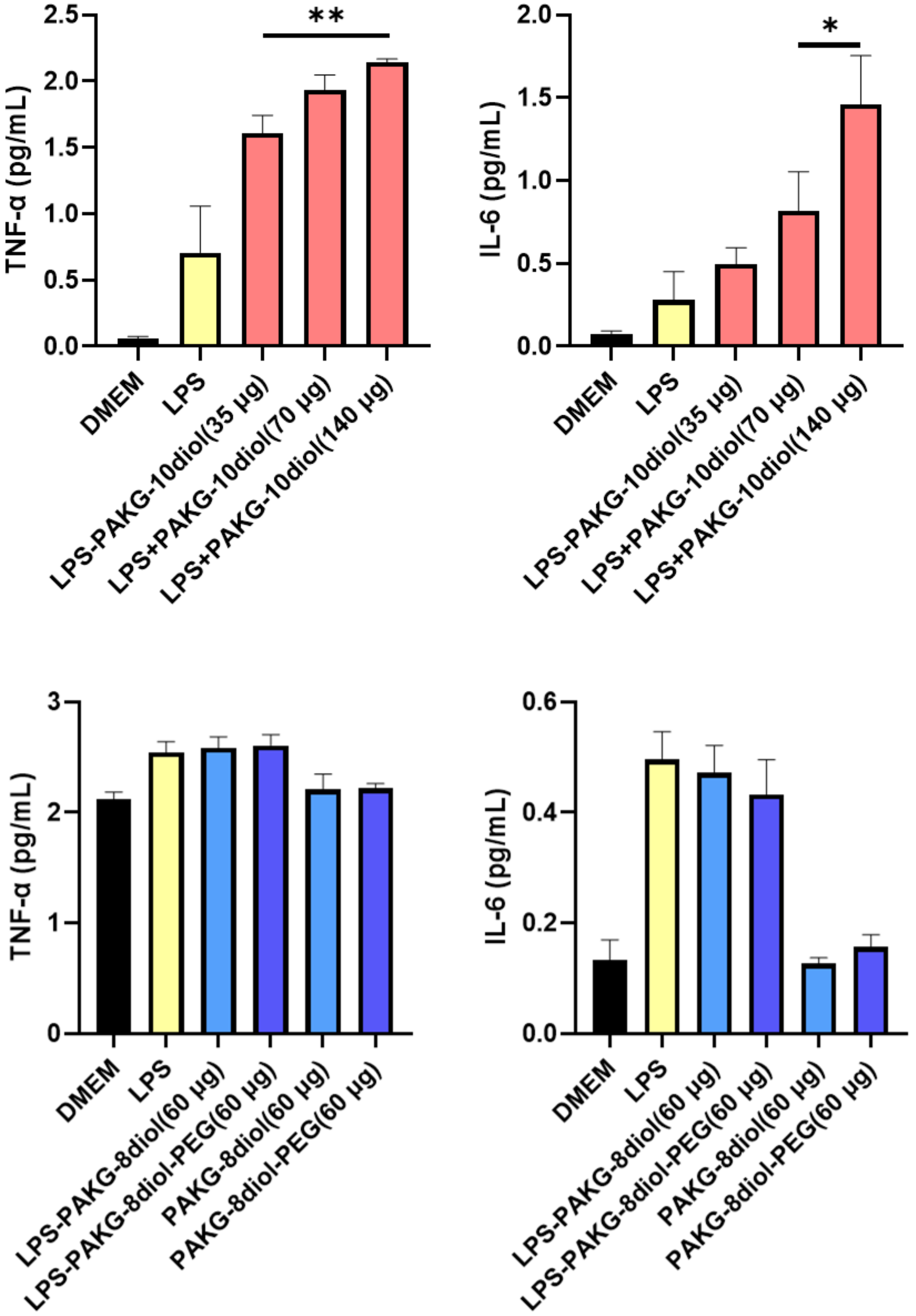

Figure S7. Elisa determination of pro-inflammatory cytokines (TNF-α, IL-6) in J774A.1 macrophages with PAKG-10diol, PAKG-8diol, PAKG-8diol-PEG. Data are expressed as mean ± SD (n = 3, *p < 0.05, **p < 0.01, ***p < 0.001, ****p < 0.0001).

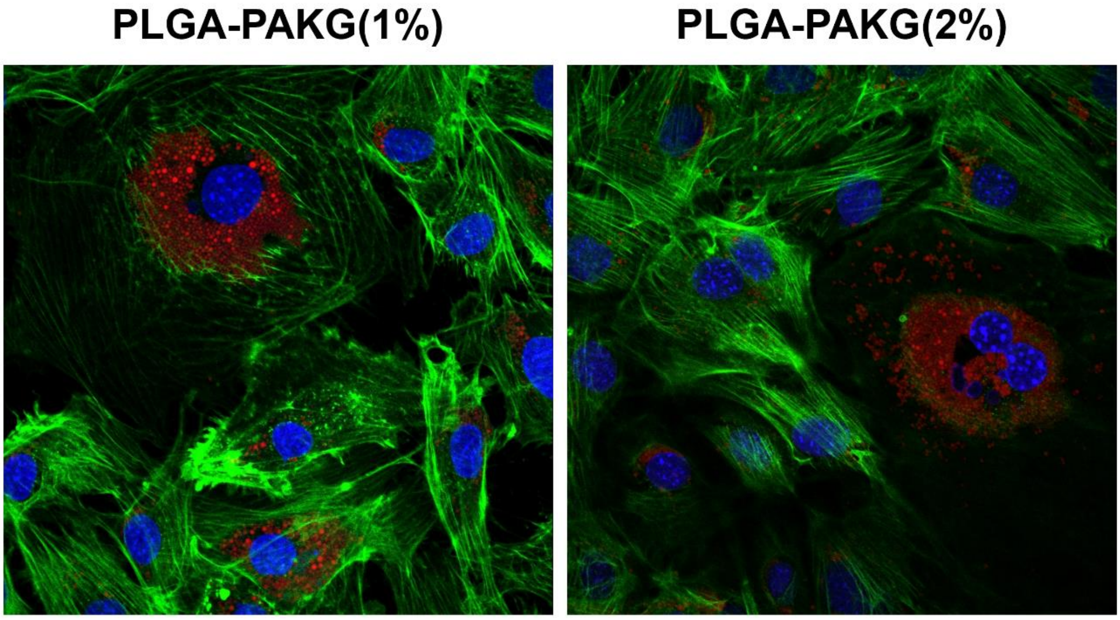

Figure S8. Confocal microscopy images of DAPI (blue)- and Phalloidin (green)-stained MC3T3-E1 cultured with PLGA-PAKG (1%) and PLGA-PAKG(2%) MPs.

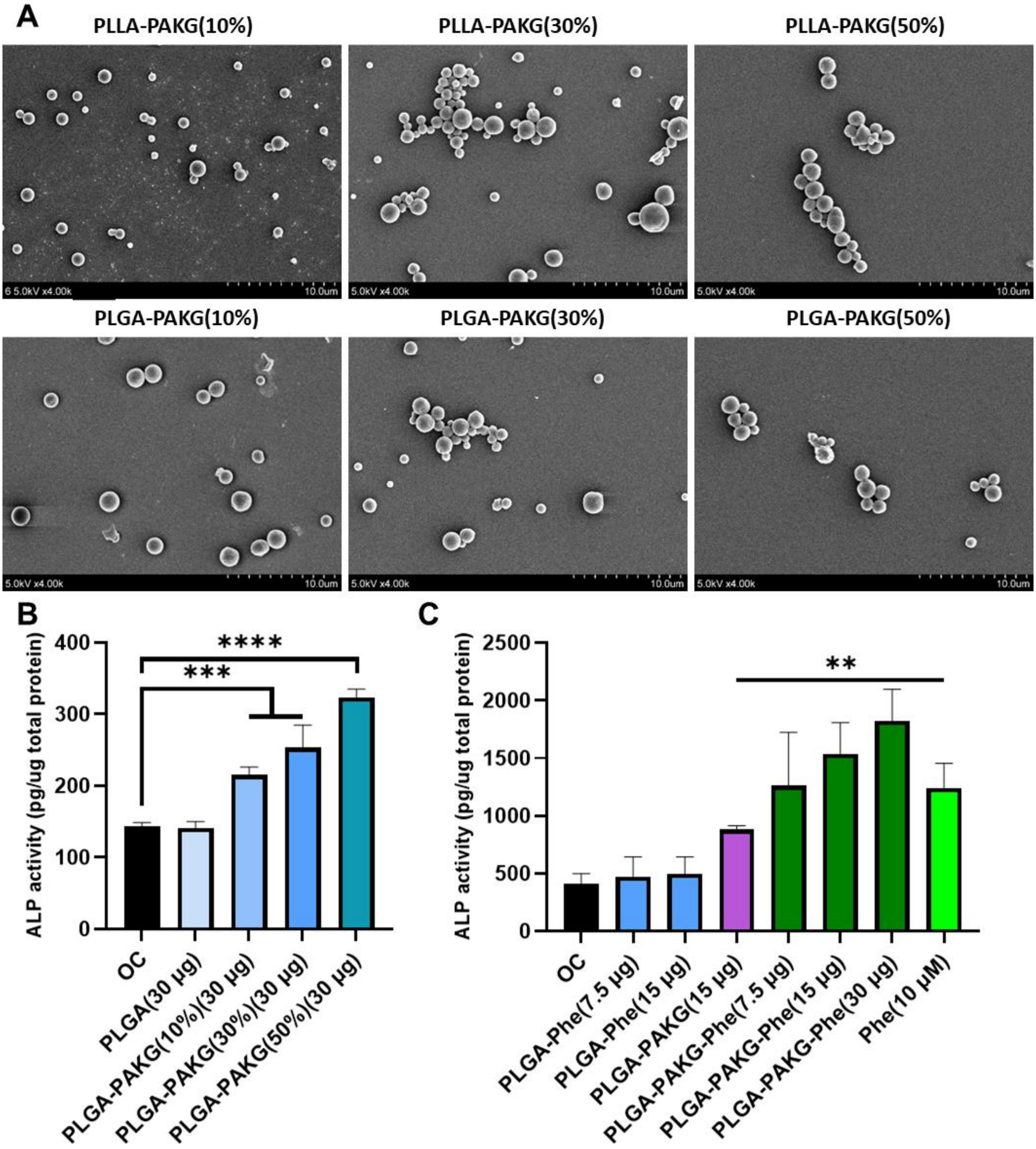

Figure S9. (A)SEM images of PLLA-PAKG (10%, 30%, 50%) and PLGA-PAKG (10%, 30%, 50%). (B) ALP activity of MC3T3-E1 with PLGA MPs containing different ratio of PAKG (0, 10, 30, 50%). (C) ALP activity of MC3T3-E1 with different doses of PLGA-Phe and PLGA-PAKG-Phe for dose optimization. Data are expressed as mean ± SD (n = 3, $*p < 0.05$, $**p < 0.01$, $***p < 0.001$, $****p < 0.0001$).

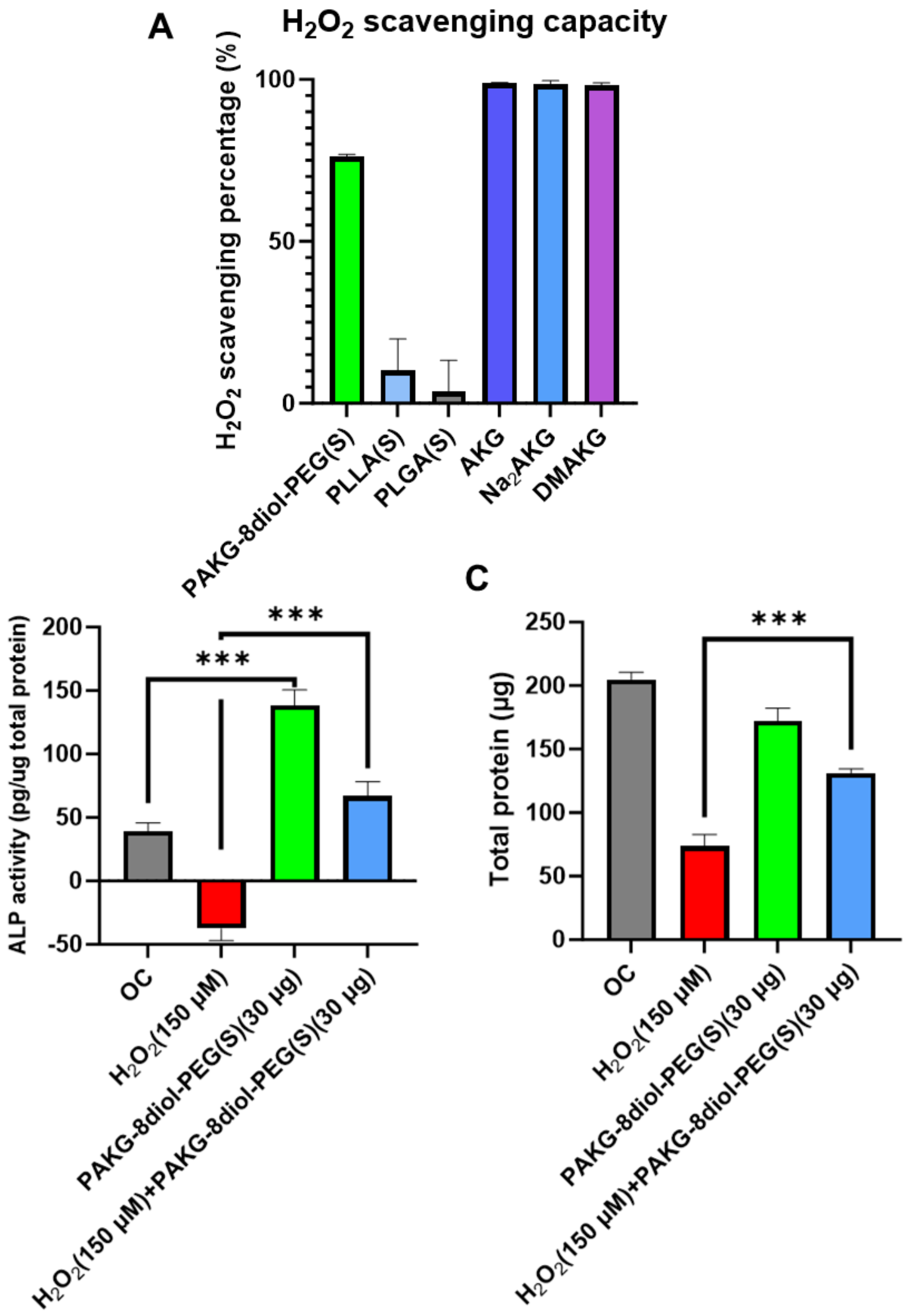

Figure S10. (A) Hydrogen peroxide (H_2O_2) scavenging capacity test for PAKG-8diol-PEG(S), PLLA, PLGA MPs and AKG, Na_2AKG, DMAKG. (B)(C) ALP activity and Total protein of MC3T3-E1 with H_2O_2 and PAKG-8diol-PEG(S) MPs to test rescuing effect of PAKG-8diol-PEG MPs in presence of H_2O_2. Data are expressed as mean ± SD (n = 3, *p < 0.05, **p < 0.01, ***p < 0.001, ****p < 0.0001).

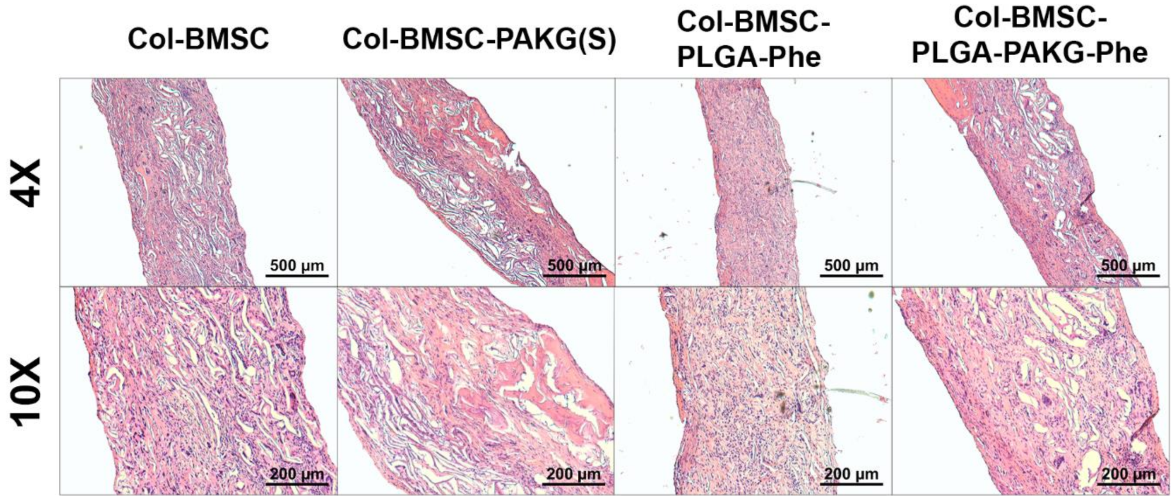

Figure S11. Representative H&E-stained tissue sections acquired from the mouse cranial defects after 6 weeks post-implantation. (Scale bars = 500 μm in the upper panel and 200 μm in the lower panel).
